# Drying kinetics and quality dynamics of ultrasound-assisted dried selenium-enriched germinated black rice

**DOI:** 10.1016/j.ultsonch.2023.106468

**Published:** 2023-06-07

**Authors:** Muhammad Tayyab Rashid, Kunlun Liu, Deng-Zhong Wei, Mushtaque Ahmed Jatoi, Qingyun Li, Frederick Sarpong

**Affiliations:** a College of Food Science and Engineering, Henan University of Technology, Zhengzhou 450001, China; b School of Food and Strategic Reserves, Henan University of Technology, Zhengzhou 450001, China; c Department of Botany, Shah Abdul Latif University, Khairpur 66020, Sindh, Pakistan; d Value Addition Division, Oil Palm Research Institute, Council for Scientific and Industrial Research, Box 74, Kade, Ghana

**Keywords:** Black rice, Drying kinetics, Selenium, Ultrasound, Anthocyanins, Volatile compounds

## Abstract

Black rice is a functional food due to its higher protein, fiber, iron, antioxidant compounds, and other health benefits than traditional rice. The ultrasonic (US) pretreatments (10, 20, and 50 min) followed by hot-air drying (50, 60, and 70 °C) were applied to study the drying kinetics, mathematical modeling, thermodynamics, microstructure, bioactive profile, volatile compounds and to lock the nutritional composition of selenium-enriched germinated black rice (SeGBR). Ultrasonic-treated samples exhibited a 20.5% reduced drying time than control ones. The Hii model accurately describes the drying kinetics of SeGBR with the highest R^2^ (>0.997 to 1.00) among the fifteen studied models. The activation energy values in US-SeGBR varied from 3.97 to 13.90 kJ/mol, while the specific energy consumption ranged from 6.45 to 12.32 kWh/kg, which was lower than untreated. The obtained thermodynamic attributes of dried black rice revealed that the process was endothermic and non-spontaneous. Gallic acid, kaempferol, and cyanidin 3-glucoside were present in high concentrations in phenolics, flavonoids, and anthocyanins, respectively. The HS-SPME-GC–MS investigation detected and quantified 55 volatile compounds. The US-treated SeGBR had more volatile compounds, which may stimulate the release of more flavorful substances. The scanning electronic micrograph shows that the US-treated samples absorbed high water through several micro-cavities. Selenium concentration was significantly higher in US-treated samples at 50 °C than in control samples. In conclusion, ultrasound-assisted hot-air drying accelerated drying and improved SeGBR quality, which is crucial for the food industry and global promotion of this healthiest rice variety.

## Highlights

• Drying efficacy of ultrasonic treatments were test first time on selenized black rice.
• Ultrasound-treated samples had greater selenium levels than untreated.
• Ultrasonic dried samples significantly decrease drying time of germinated black rice.
• The Hii model best fitted the drying kinetics of selenium enriched black rice.
• Sonicated samples improved phenolic, anthocyanin, and volatile compound profiles.

## 1 Introduction

Black rice originated from China and is mainly cultivated in Southeast Asian countries, and it is the healthiest variety of rice. Black rice is also a functional food abundant in health-promoting flavonoids, polyphenols, essential secondary metabolites, anthocyanins, and antioxidants, which can prevent the development of cancer cells, atherosclerosis, hypertension, diabetes, osteoporosis, asthma, gastrointestinal issues, etc. [1]. Despite its numerous nutritional qualities, black rice is an undervalued rice crop due to its high price and expected market availability [2].

Selenium is a vital human element in biological processes such as antioxidant content and immunological modulation [3]. Inadequate selenium levels can lead to various health issues, such as Kashin-Beck disease and Keshan disease [4]. In regions such as Tibet, where the soil lacks sufficient selenium, the population is greatly affected and has poor health. It is, therefore, essential to find effective methods for increasing selenium levels. Germination is a conventional technique for enhancing the nutritional content of grains. Germination may improve the concentration of folic acid, γ-aminobutyric acid, polyphenols, and other physiologically active components in grain [5]. In addition, germination is an efficient method for selenium accumulating in grain. A previous study found that brown rice can get selenium during germination, primarily in selenium-containing proteins [6]. The study also found that selenium-containing proteins purified from germinated brown rice that had been selenized (Se-GBR) had intense antioxidant activity and could be utilized as effective antioxidants. Numerous other Se-enriched foods, such as rice grain [4], brown rice [6], green tea [7], and Zea mays [8], have acknowledged that Se exerts powerful effects on particular intracellular selenoproteins and potent antioxidant activities. It would be interesting to investigate whether ultrasonic treatment of dried black rice improves its nutritional qualities while maintaining its selenium content, as there is currently no such research.

Drying is an economical and efficient way of preserving grains for lengthy periods. Hot-air drying is the most common drying method, but it is time-consuming. Our previous research has demonstrated that hot-air drying may reduce sample quality by causing enzymatic browning and shrinkage and a loss in rehydration and phenolic chemicals [9], [10]. In addition, sweet potato microstructure was significantly altered during hot-air drying [11]. To address the limits of conventional drying techniques, enhanced ultrasonic hot-air-drying methods are often utilized to reduce drying time and increase product quality [12], [13]. Ultrasonic technology involves using mechanical waves to create ultrasonic cavitation, which leads to increased particle movement and modification of the material's internal structure. This results in improved water diffusion and enhanced dehumidification through heat energy. The impact of high-intensity ultrasound on drying kinetics is predominantly driven by mechanical factors [14]. Fresh fruits and vegetables are commonly dried using ultrasonic technology to maintain product quality. A study conducted by Yang et al. and Zhang et al. [15], [16] investigated the effects of ultrasonic treatment on the metabolomics of soybean seeds and peanuts and discovered significant changes in the levels of amino acids, organic acids, and sugars, as well as increased levels of several antioxidants such as glutathione and vitamin C. However, the application of ultrasonic technology in the drying process of germinated black rice seeds is not yet established.

Rice is valued for its flavors, nutrient benefits, and other characteristics. Rice volatiles is one of rice's primary features, significantly influencing rice quality. However, the drying process is rigorous, and rice volatiles may vary over time owing to oxidation and mass loss. Since flavor is a crucial aspect of excellent freshness [17], various research has demonstrated that its chemical content is assumed to be essential for its quality. The method of headspace solid phase microextraction (HS-SPME) is a commonly used approach for analyzing volatile compounds in food and beverage products. This is due to its ease of use, versatility, quick sample preparation, and sensitivity. Additionally, the combination of gas chromatography and mass spectrometry (GC–MS) has significantly reduced both quantitative and qualitative analysis times [6], [18]. However, few systematic studies imply SeGBR quality changes after drying or identify SeGBR phenolic compounds and anthocyanins with taste characteristics.

The purpose of the study was to analyze the impact of various drying temperatures (50, 60, and 70 °C) and ultrasonic durations (10, 20, and 50 min) on the drying attributes, mathematical modeling, thermodynamics, energy consumption, microstructure, selenium concentration, bioactivity, and anthocyanin profile of germinated black rice (GBR). The flavor of Se GBR treated with ultrasound was analyzed using HS-SPME and GC–MS. This research may help to give theoretical guidelines for ultrasonic enhanced grain hot air drying.

## 2 Material and methods

### 2.1 Raw material collection

Rough black paddy rice with a high germination rate (>85%) of variety Yanghei No. 3, obtained from the Henan Academy of Agricultural Science (China), was used in this study. The black rice was obtained directly from rough rice hulled with a machine. The broken rice was separated by a broken rice separator (FQS-13X20, Taizhou grain instrument Co., Ltd. China).

### 2.2 High-intensity power ultrasound (HIPU)

Sanitization of the samples (360 g) was accomplished before the application of ultrasound by soaking them in 0.1% sodium hypochlorite (500 mL) for 30 min and then washed twice with deionized water. Using an ultrasonic irradiator (Tianhua Co. located in Jining, China), the sterile grains were subjected to ultrasonic stimulation at 40 kHz with an acoustic power of 500 W/cm3 for a holding time of 10, 30, and 50 min.

### 2.3 Germination process

After the ultrasonic treatment, the samples germinated in the dark with 60 μM sodium selenite at 25 °C for 60 h. After germination, the sample was washed with ultrapure water. Samples without ultrasonic treatments and selenite solution were used as the control group. Selenium-enriched black rice was prepared following a described method [19].

### 2.4 Convective hot-air drying (HAD)

The samples were dried in a hot-air dryer with a temperature (of 50, 60, and 70 °C) and 1.5 m/s air velocity. Each 15 g sample was dried until a uniform constant weight was obtained. After a 10-min interval, sample weights were taken until the required moisture content of 5.01.0% was not obtained.

### 2.5 Drying kinetics and mathematical modeling

The experimental result of the study was combined with the 15 drying equations presented in supplementary Table S1. In addition, the following Eqs. (1), (2) were used to calculate the moisture ratio (MR) and drying rate (DR) during drying experiments.

(1) $$\mathrm{MR}=\frac{M_{1}-M_{e}}{M_{o}-M_{e}}$$

(2) $$\mathrm{DR}=\frac{M_{d+d_{1}-M_{t}}}{d_{t}}$$

### 2.6 Non-linear regression analysis

Four statistical functions were used to measure the good fit between the modeling and the experimental data (R^2^, RMSE, RSS, and reduced χ^2^). A maximized R^2^ and minimized RSS, RMSE, and χ^2^ values indicate a better model fit.

### 2.7 Activation energy and thermodynamics

(3) $$D=D_{0}e^{\frac{E_{a}}{R_{\mathrm{gT}}}}$$

where *E*_a_ is defined as the activation energy (kJ/mol), *R*_g_ is the universal gas (*R*_g_ = 8:31451 J/mol/K), and D_0_ is an integration constant (m^2^/s).

(4) $$\Delta H=E_{a}-RT$$

(5) $$\Delta S=R[\ln\left(D_{0}\right)-\ln\left(\frac{K_{b}}{h_{p}}\right)-\ln\left(T\right)]$$

(6) ΔG=ΔH-TΔS

### 2.8 Total energy consumption calculation (ET_HA_)

The total energy consumption (ET_HA_) in a convection dryer was defined as the total electrical energy applied to determine the blower (E_b_) and the electric energy heater (E_e_) during the drying process. The energy consumption by the heaters was determined using Eq (7):

(7) $$E_{\in }=A\times v\times P_{a}\times C_{a}\times \Delta T\times t$$

Energy consumption designed applying (8):

(8) $$E_{b}=\frac{V^{3}}{16600}$$

### 2.9 Quality attributes

#### 2.9.1 Preparation of extract

Samples (2.5 g) were sonicated (40 kHz) in an ultrasonic cleaner at 60 °C for 60 min after being dissolved in 5 mL of 68% (v/v) aqueous ethanol. A rotary evaporator operating at 60 °C was used to extract the solvent after the mixture had been centrifuged at 4,000 rpm for 10 min. The extracts were stored at −25 °C in a brown bottle until further examination.

#### 2.9.2 HPLC analysis of phenolics, flavonoids, and anthocyanins.

HPLC analysis of phenolic acids and flavonoids was performed using a Waters HPLC (model E2695) with a UV–visible photodiode array detector. The HPLC analysis was conducted following the protocol fully described by Peanparkdee et al. [20].

### 2.10 Isolation and identification of volatile compounds

#### 2.10.1 HS–SPME/GC–MS analysis

2.5 g of each sample was placed in 20 mL glass vials with screw-on covers and sealed with septa. The sample (5.00 g) was mixed with 100 μL of the internal standard (3-octanol at a concentration of 50 ng/mL in distilled water) and 5.00 g of sodium sulfate during the sampling process. Volatile chemicals were extracted and absorbed using SPME fibers (50 μm DVB/CAR/PDMS from Supelco, Pennsylvania, USA) at 60 °C for 30 min. Using an automatic autosampler, the fiber-bearing volatile compounds were immediately injected into the GC inlet and desorbed for 5 min at 250 °C. According to our earlier study by Liu et al., the gradient program and data processing were followed (2016). We used the gradient program for this experiment and processed the data described in our earlier work with Li et al. [6].

### 2.11 Microstructure analysis

The changes during the drying were microstructurally examined using Scanning Electron Microscope (a JEOL model JSM 5800LV) at 5 kV. The samples were coated with gold in a Sputter Coater (BALZERS, model SCD050).

### 2.12 Statistical analysis

Each experiment was conducted in triplicate. Statistical analysis was performed using Origin Ro 2018, and ± SD was determined by Tukey's tests at p < 0.05.

## 3 Results and discussion

### 3.1 Drying kinetics curve

**Fig. 1:**
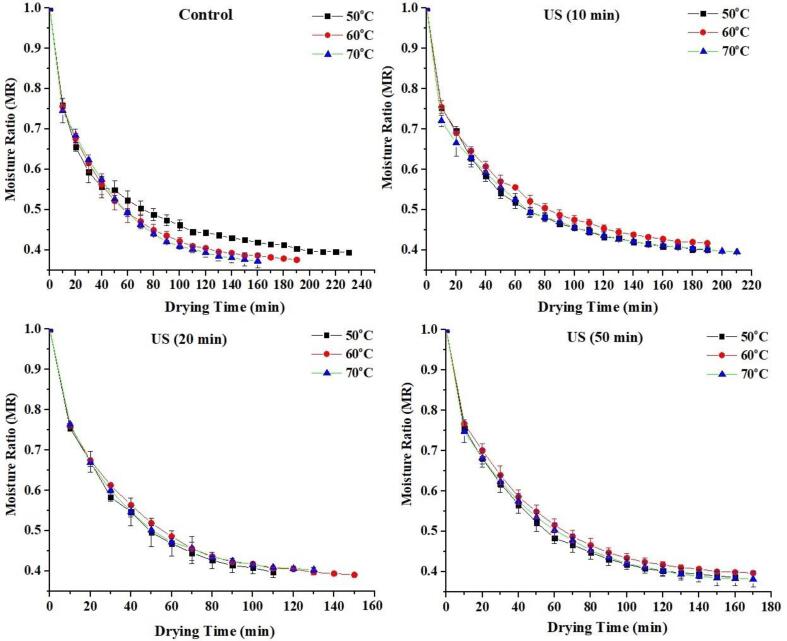
Effect of ultrasound and drying temperatures on moisture loss of SeGBR.

The drying kinetic curves of selenium-enriched germinated black rice (SeGBR) at various ultrasonic times and drying temperature is depicted in Fig. 1. The moisture content of germinated rice decreased rapidly during the first 20 min of drying, and the rate of moisture loss was the same for all treatments. This might be because the moisture in the samples at this early stage was mostly on the surface of the samples, where it could quickly vaporize and cause similar drying rates regardless of the sample type [21]. However, after this period, there was a significant difference in the moisture, with SeGBR treated in the US (20 min) exhibiting a lower moisture content than the US at 10 and 20 min in control samples at all drying temperatures. The US (20 min) ultrasonically treated group had the fastest sample drying time at 50 °C (p < 0.05) than other US treated and control sample groups. Ultrasound may induce rapid expansion and contraction of plant cells, which could develop air bubbles in and around the sample. As a result, sizeable transient pressure variations cause changes in plant cell viscosity and surface tension, as well as the formation of microchannels, thereby decreasing the drying time [9]. The findings of this study are comparable to the drying kinetics of germinated barley seeds dried by ultrasonic-assisted hot-air drying [3]. The drying temperature of SeGBR is likewise affected by ultrasonic time. As shown in Fig. 1, when the ultrasonic duration is 20 min, and the drying temperature is 50, 60, or 70 °C, the ultrasonic group requires 110, 130, or 150 min of drying time, respectively.

In comparison to the control group, drying time was decreased by 20.5%. Thus, ultrasound in the drying process can speed up the moisture distribution within the sample, thereby reducing the drying time and increasing the drying rate. The ultrasound waves produced by the radiation disk can penetrate directly into the material when the sample is placed on an ultrasonic radiation disk (HAD). Ultrasound has high frequency vibrations that dramatically and repeatedly stretch and contract the microstructure of samples, creating many microbubbles within the materials. Blasting those bubbles instantly could generate kinetic and compressive energy [22].

### 3.2 Drying rate curves

**Fig. 2:**
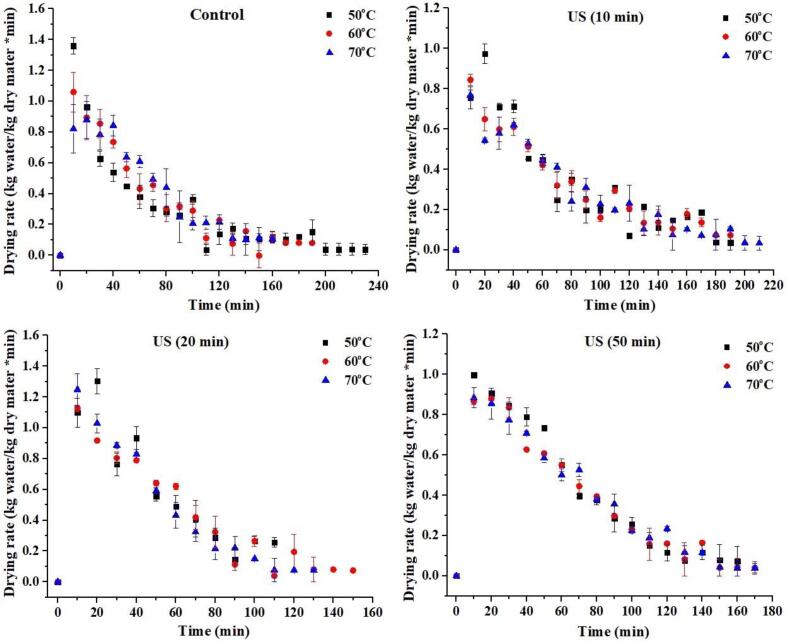
Effect of ultrasound and drying temperatures on drying rate of SeGBR.

The changes in drying rate versus drying time are depicted in Fig. 2. The drying rate decreased as the process progressed under various drying temperatures and ultrasound timings, and no constant rate period was noticed. At 10-, 20-, and 50-min US timing, the treatment groups had 9.45, 21.5, and 28.5% higher initial drying rates than the control group. In contrast, the 20-min ultrasound group at 60 °C and 70 °C dried faster. This could be because germinated black rice experiences an enormous heat transfer homogenous mass drive when subjected to high ultrasonic power (500 W), which causes significant internal heat production due to the high energy absorption of the grain layer (GBR) [3]. However, drying rates vary significantly at different temperatures due to an imbalance in the vapor pressure differential between the grain's center and surface during drying, which causes unstable moisture transport from the inner core to its surface [23]. The results showed that increasing ultrasonic intensity in hot-air drying led to a higher SeGBR drying rate. Applying ultrasonic waves to a solid/gas system causes variations in oscillation speed, microfluidics, and pressure at the interface, resulting in mechanical agitation of the gas medium. This agitation facilitates the transfer of water from the sample surface to the air, thus accelerating the drying process [24].

Fig. 2b shows that all processed GBR drying rates started with a longer drying time and then gradually decreased. Similar drying rates have been found for microwave drying of apple pomace [25], apple slices [26], and grapes [27], demonstrating that the warm-up time is minimal and the decrease rate is relatively lengthy. The rapid drying rate at the start of drying might be ascribed to the vast quantity of free water inside the GBR grain and the significant ultrasonic absorption capacity, resulting in a massive driving force for water transfer. Furthermore, once the maximum drying rate is achieved, the drying process fully enters all treatment rate decrease phases. It demonstrates that the SeGBR hot-air drying process may be regarded as a falling rate owing to a rapid warm-up rate during the drying phase. In general, the drop rate period dominates the drying process for most fruits and vegetables, where moisture transport is limited by internal heat and mass transfer resistance [28], [29].

### 3.3 Mathematical modeling of SeGBR

**Fig. 3:**
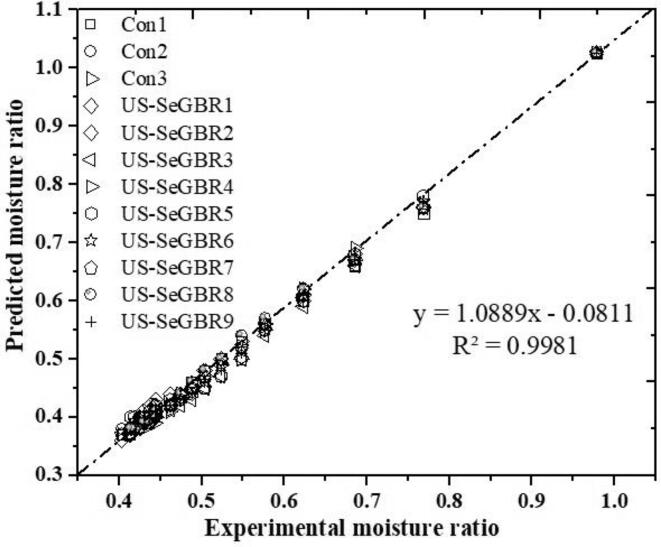
Validation of the Hii model by comparing black rice's predicted vs. experiment data.

The drying kinetics of selenium-enriched germinated black rice during hot-air drying were described using drying models. The results of fitting the specified mathematical layer model to the experimental moisture data are depicted in Table 1. Mathematical models can help us predict the drying effect through statistical analysis. Even though all the selected 15 models fitted satisfactorily with the experimental data, the Hii model is said to effectively describe the drying kinetics of GBR with the highest R^2^ (>0.997 to 1.00), lowest RMSE (<0.0027 to 0.0062) and lowest RSS (<0.00) for all drying conditions (Table 1). Fig. 3 displays the efficacy of the Hii model via a comparison of the predicted humidity data with the experimental data. Therefore, the Hii model was used to estimate moisture rate data under various drying settings, followed by linear regression, demonstrating the model's adaptability to represent the hot-air drying behavior of black rice. According to Le & Jittanit [30], the Hii model predicts jasmine brown rice's drying kinetics well. Similar results have been reported about using Hii models to explain sweet potatoes' drying performance [31]. In addition to providing relatively accurate quantifications, model fitting could be used to compare drying kinetics under different conditions, providing detailed information about drying time, rate, sensitivity, and resistance to different treatments during the whole drying process [32].

**Table 1:** Averages of selected models fitted to the ultrasound-assisted hot-air drying for selenium-enriched germinated black rice.

Sample Codes	T (°C)	Coefficients	R^2^	RSS	χ^2^	RMSE
*Hii Model*						
Con1	50	a. 0.532, k1. 0.008, n. 0.689, b. 0.469, k2. 0.138	0.999	0.00	2.77 × 10^−05^	0.0035
Con2	60	a. 0.065, k1. −0.055, n. 0.596, b. 0.934, k2. 0.080	1.000	0.00	1.63 × 10^−05^	0.0027
Con3	70	a. 0.000, k1. −0.366, n. 0.540, b. 0.998, k2. 0.078	0.998	0.00	0.000111	0.0062
US-SeGBR1	50	a. 0.073, k1. −0.060, n. 0.555, b. 0.926, k2. 0.089	0.999	0.00	4.96 × 10^−04^	0.0052
US-SeGBR2	60	a. 0.010, k1. −0.143, n. 0.579, b. 0.990, k2.0.074	0.999	0.00	4.37 × 10^−05^	0.0042
US-SeGBR3	70	a. 0.994, k1. 0.070, n. 0.609, b. 0.006, k2. −0.168	0.999	0.00	2.45 × 10^−05^	0.0047
US-SeGBR4	50	a. 9.89 × 10^−5^, k1. −0.617, n. 0.445, b. 0.999, k2. 0.099	0.999	0.00	2.96 × 10^−05^	0.0036
US-SeGBR5	60	a. 0.003, k1. −0.208, n. 0.563b. 0.996, k2. 0.070	0.999	0.00	4.93 × 10^−05^	0.0049
US-SeGBR6	70	a. 0.010, k1. −0.135, n. 0.598, b. 0.989, k2. 0.070	0.999	0.00	3.37 × 10^−05^	0.0029
US-SeGBR7	50	a. 3.46 × 10^–3^, k1. −0.830, n. 0.410, b. 0.989, k2. 0.120	0.998	0.00	4.66 × 10^−05^	0.0048
US-SeGBR8	60	a. 0.000, k1. −0.414, n. 0.516, b. 0.999, k2. 0.084	0.999	0.00	4.37 × 10^−05^	0.0049
US-SeGBR9	70	a. 0.119, k1. −0.031, n. 0.666, b. 0.881, k2. 0.073	0.997	0.00	3.06 × 10^−05^	0.0036
*Modified Midilli model*						
Con1	50	a. 0.638, k. 0.140, n. 0.565, b. 0.363	0.998	0.00	8.98 × 10^−05^	0.0641
Con2	60	a. 0.649, k. 0.089, n. 0.690, b. 0.349	0.999	0.00	5.29 × 10^−05^	0.0048
Con3	70	a. 0.693, k. 0.089, n. 0.654, b. 0.304	0.997	0.00	0.000204	0.0084
US-SeGBR1	50	a. 0.637, k. 0.105, n. 0.630, b. 0.362	0.998	0.00	7.18 × 10^−05^	0.0062
US-SeGBR2	60	a. 0.650, k. 0.085, n. 0.701, b. 0.347	0.998	0.00	7.10 × 10^−05^	0.0054
US-SeGBR3	70	a. 0.662, k. 0.085, n. 0.717, b. 0.337	0.999	0.00	2.45 × 10^−05^	0.0047
US-SeGBR4	50	a. 0.673, k. 0.123, n. 0.540, b. 0.326	0.999	0.00	5.27 × 10^−05^	0.0048
US-SeGBR5	60	a. 0.648, k. 0.080, n. 0.693, b. 0.349	0.998	0.00	0.000118	0.0076
US-SeGBR6	70	a. 0.643, k. 0.080, n. 0.728, b. 0.355	0.998	0.00	0.000115	0.0053
US-SeGBR7	50	a. 0.687, k. 0.148, n. 0.505, b. 0.311	0.997	0.00	0.000100	0.0070
US-SeGBR8	60	a. 0.682, k. 0.096, n. 0.636, b. 0.315	0.998	0.00	0.000103	0.0075
US-SeGBR9	70	a. 0.632, k. 0.082, n. 0.741, b. 0.368	0.997	0.00	5.98 × 10^−05^	0.0050

Con = Control; US-SeGBR = Ultrasound selenium-enriched germinated black rice.

### 3.4 Activation energy (Ea)

The activation energy (Ea) is crucial in determining the minimum energy required for moisture diffusion within a material. The results of the activation energy in the untreated and ultrasonic-treated SeGBR drying process are shown in Table 2. The activation energy increased from 3.97 kJ/mol to 13.90 kJ/mol for samples treated with ultrasound for 10, 20, and 30 min, respectively. Comparing the activation energies of ultrasound-treated and untreated samples revealed an increase, whereas the control group requires significantly more energy, 20 Kh/mol, to initiate the drying process, as shown in Fig. 1 and Fig. 2. This increase suggests that ultrasound-treated SeGBR needs more energy to start diffusion. The temperature sensitivity of the process could account for the rise in activation energy, indicating that sonicated samples are more prone to diffusion than controls. The activation energy of agricultural products varies, ranging from 12.7 to 110 kJ/mol [33]. Rashid et al. [34] discovered a similar range of 11.03 to 57.42 kJ/mol while examining the effects of various multi-frequency ultrasonic waves on the infrared drying of sweet potatoes. The activation energy for drying wheat increased as the heating rate increased. At a heating rate of 2 C/min, the activation energy was 14.76 kJ/mol, while it rose to 28.17 kJ/mol at a heating rate of 10 C/min [35]. Although the ultrasound is much shorter than we used, the activation energy values are comparable to our work.

**Table 2:** Effect of ultrasound-assisted hot-air drying on selenium-enriched germinated black rice regarding activation energy, thermodynamics, and specific energy consumption.

Sample codes	Ultrasonic time (min)	Temperature (^o^C)	Activation Energy (kJ/mol)	R^2^	ΔH(kJ/mol)	ΔG(kJ/mol)	ΔS(kJ/mol)	Energy Consumption (kWh/kg)
Con1	**Untreated**	50	20.00	0.9989	19.58	173.22	−475.42	13.49
Con2		60			19.50	179.31	−479.70	11.15
Con3		70			19.42	185.34	−483.54	11.15
US-SeGBR1	**10**	50	11.93	0.9945	11.52	173.87	−502.40	12.32
US-SeGBR1		60			11.44	179.66	−504.95	11.15
US-SeGBR1		70			11.35	185.54	−507.62	9.39
US-SeGBR1	**20**	50	3.97	0.9984	3.56	174.00	−527.44	10.56
US-SeGBR1		60			3.47	179.89	−529.53	9.97
US-SeGBR1		70			3.39	185.19	−529.78	9.39
US-SeGBR1	**50**	50	13.80	0.9953	13.39	173.48	−495.40	8.80
US-SeGBR1		60			13.30	179.68	−499.41	7.63
US-SeGBR1		70			13.22	185.24	−501.29	6.45

Con = Control; US-SeGBR = Ultrasound selenium-enriched germinated black rice.

### 3.5 Thermodynamics properties

The thermodynamic properties studied in this investigation were primarily linked to forming an intermediate product, also known as a transition state or activated complex. The thermodynamics of drying can be fully explained by the thermo-kinetics of drying, where specific entropy is related to the amount of energy needed to remove the water absorbed by the product [36], [37]. The drying process's specific enthalpy (H) reduced marginally in both untreated and ultrasound-treated SeGBR samples. However, raising the drying temperature led to a decrease in enthalpy. The enthalpy values recorded were positive, in line with the findings of Rashid et al. [34] in their study of drying sweet potatoes. This study demonstrates that an endothermic reaction occurs when the activated complex (PD - H2O) is produced, proving that the physical and chemical conversion needs heat [38], [39]. According to Al-Zybaidy & Khalil [40], ΔH indicates the difference between the active state and the reactant; hence it must be positive. The endothermic reaction in this study suggests that the addition of energy at drying temperature or ultrasound may have aided activation complex formation. The study found that the energy required for the reaction (enthalpy of activation) in samples treated with sonication was lower than in untreated black rice. This suggests that ultrasound combined with different temperatures can supply the necessary energy to form the activated complex, resulting in faster moisture absorption, as demonstrated in Fig. 1, Fig. 2.

In contrast, the value of entropy (S) in untreated and sonicated selenium-rich germinated black rice is negative. Entropy is often described as the degree of the disorder [41]. Our study showed that the modulus entropy of sonicated samples was greater than that of untreated samples. This suggests an increase in the system order, which is against entropy's nature of leading to disorder. The molecular structure, in this instance, became more organized [39]. H-bonds formed between water and other substances are believed to be more structured during hydration than H-bonds within the water. Ultrasound measurements from US-RBSe1 to US-RBSe9 showed higher entropy values compared to control samples Con1 to Con3. This could be because the ultrasonic technology causes chaotic and turbulent movement in the molecules, hindering the formation of an organized molecular structure during activation. Therefore, a more significant reduction in entropy was required for the process to form the activated complex to achieve this organization. The organization faces difficulties with ultrasound processing as the sonolysis produced by cavitation creates free radicals in water. A similar effect was observed during rice parboiling, showing that entropy was negative and constant with temperature, indicating no significant increase in disorder in the system [42]. Jideani & Mpotokwana [43] found that the entropy of Botswana Bambara groundnut varieties decreased during hydration, demonstrating an increase in order within the system, resulting in a less random arrangement. In addition, the drying temperature increase led to a rise in the process entropy. Rashid et al. [34] also reported this temperature effect for sweet potatoes.

The Gibbs free energy (ΔG) of untreated and ultrasonic-treated black rice is positive, indicating that the reaction is not spontaneous. Temperature and ultrasonic levels have the most negligible impact. The drying temperature causes an increase in Gibbs free energy, which is identical to the rise in ultrasonic-dried samples at 50, 60, and 90 °C. The outcomes are comparable to those of Miao et al. [38] regarding common beans. Borges et al. [44] state that the ΔG value measures the work that promotes grain moisture. A positive Gibbs free energy value implies that the drying process is exothermic, needing heat from the environment. However, the rise due to ultrasonic processing shows that less heat is necessary for water breakdown. Nadi & Tzempelikos [39] found similar results during the vacuum drying of apples, and Souza et al. [45] observed the same trend in the dehydration of mesocarp in pequi or souari nut (Caryocar brasiliense). This study's results show that the black rice drying process is not spontaneous and requires additional energy from the environment.

Furthermore, the high molecular organization was noted during this transition. Miano et al. [38] also found that the thermodynamic properties of the activated complex exhibit low-temperature dependence, which was confirmed in our research. Our findings indicate that increasing the temperature does not influence the spontaneous formation of the activated complex, the degree of molecular organization or the energies involved.

### 3.6 Specific energy consumptions (SEC)

The SEC was established as the total energy required to reduce the moisture content of germinated black rice to 10 ± 0.5 kg of water per kg of dry matter. Table 2 displays the mean SEC values for control and US-dried SeGBR under various treatment conditions. The SEC value varies depending on the drying conditions. For both untreated and ultrasonic-treated SeGBR, an increase in drying air temperature decreased SEC values. An increase in the temperature difference between the ambient air and the drying air, as well as a decrease in the hot-air blower's power consumption, likely led to the observed result [46]. High air temperatures create more water vapor pressure within the kernels, leading to the release of moisture. However, this increase in energy usage also produces a higher amount of water vapor during the drying process. As a result, the diffusion coefficient grows, and the effective conductance (EC) falls [47]. Similar outcomes have been observed for the air ultrasonic drying of corn husks when the energy required for drying air exceeds the energy saved due to the reduction in drying time [48]. In addition, ultrasonic times of 20 and 50 min can shorten the total drying time, reducing energy usage. According to the results above, an increase in ultrasonic processing time can achieve products with a high rehydration ratio and an excellent appearance. However, high ultrasound power (500 kW) can result in increased ultrasound energy loss and adverse tissue damage; therefore, ultrasound with 500 kW high power and longer ultrasound timing (20 & 50 min) is suitable for accelerating the SeGBR drying process. Higher drying temperatures result in shorter drying times, lower operating costs, and better quality, but they can also cause higher nutrient degradation rates, resulting in inappropriate damage. As a result, a drying temperature of 50–70 °C is appropriate for achieving an increased drying rate while maintaining product quality. Similar results were obtained in rough rice using convection drying assisted by air-borne ultrasound [49].

### 3.7 Individual phenolics compounds

The contents of individual phenolic compounds in the selenium-enriched germinated rice acquired from black rice and control samples were determined using HPLC-PDA. Due to the lack of available standards, only 11 phenolic and 3 flavonoid compounds and 6 anthocyanidins were quantified.

The phenolic acid profile of SeGBR was found to contain high levels of ellagic acid, gallic acid, Isoferulic acid, protocatechuic acid, and ellagic acid. Among these compounds, gallic acid was the most abundant in all samples of black rice. This result aligns with previous studies conducted by [50]. While gallic and ellagic acid were reported as the most abundant phenolic compounds by Hrnčič et al. [51]. As shown in Table-3, the US treatment significantly increased the extractability of almost all phenolic compounds compared to the control, while the concentrations of ferulic acid, p-coumaric acid, glucosic acid, and vanillic acid remained unchanged. Therefore, the ultrasound may induce the fragmentation of conjugated phenolic acids into accessible form, resulting in increased extractability of the samples examined here. High polyphenols extraction yields can be related to US-induced cell membrane rupture, which facilitates phenolics extraction from plant cells [52]. The highest concentration of gallic acid found in the US-SeGBR4 ultrasound-treated sample was 2605.94 µg/g, followed by 1455.15 µg/g in US-SeGBR6. Protocatechuic acid showed the second highest amount, ranging from 908.42 to 115.22 µg/g. The drying process significantly impacted the levels of individual phenolic compounds. The effects on the metabolomics of peanut kernels were explored by Talcott et al. [53], who discovered substantial changes in the levels of amino acids, organic acids, and sugars, as well as higher amounts of various phenolic compounds. Some phenolic components, such as ferulic acid, hydroxybenzoic acid, p-coumaric acid, and vanillic acid treated with sonication, were significantly lower in all drying treatments compared to control samples. However, the amounts of phenolic compounds such as ferulic acid in US-SeGBR1, US-SeGBR5, US-SeGBR7, and US-SeGBR8 samples were not identified in ultrasonic treated dried samples. The study established the following order of total phenolic acids in the ultrasound-treated samples of black rice: US-SeGBR4 (4554.75 µg/g) > US-SeGBR6 (2751.43 µg/g) > US-SeGBR3 (2471.27 µg/g) > US-SeGBR2 (2377.99 µg/g) > US-SeGBR1 (2193.92 µg/g) > US-SeGBR7 (1404.82 µg/g) > US-SeGBR5 (1380.23 µg/g) > US-SeGBR7 (1117.96 µg/g). Drying at higher temperatures is more likely to result in the loss of conjugated polyphenolic compounds, as evidenced by the fact that the content of phenolic acid is highest at 50 °C, lower at 60 °C, and highest at 70 °C. Organic edible seed germination and sprouting results in nutritional sprout products that are good alternatives to traditional plant foods. Recent research has confirmed that energy metabolism plays an important regulatory function in the postharvest preservation of fruits and vegetables. Previous research demonstrated that a positive energy status was critical for improving the production of nutritional chemicals such as phenolics [54]. Similar results were obtained in the germination of barley seeds using ultrasound-assisted drying [3].

Kaempferol was the most prevalent flavonoid, followed by quercetin; rutin was present in all samples but at low concentrations (both control and ultrasound-treated). These flavonoid compounds in black rice husks are advantageous because they have antipyretic, analgesic, anti-inflammatory, antiarthritic, antioxidant, and immunomodulatory activities [1]. The highest concentrations of kaempferol (62.39 g/g) and quercetin (49.88 g/g) were found in the US-SeGBR7 and US-SeGBR8 samples, respectively. Kaempferol is typically less prevalent than gallic acid in most products [50]. There was a marginal gain in sonicated samples relative to flavanol controls. The increase could be attributed partly to the increased extractability of flavanols in dry soft tissues and the release of phenolic compounds bound to the cell wall [55]. In addition, the quercetin content of all treatments extracted with methanol is higher than that of those removed with water (22.1 g/g) [56]. The same results were found when comparing the number of flavanols with the ultrasonic-assisted extraction of phenols from Chinese wild rice [57].

### 3.8 Anthocyanin contents

A comparative study was conducted to ascertain the impact of ultrasound on selenium-rich black rice. A total of six anthocyanins were quantified, with only five detected: cyanidin-3-galactoside, cyanidin-3-glucoside, cyanidin-3-diglucoside, cyanidin-3-Rhamnoglucoside, and malvidin-3-galactoside. Similar results were found by Goufo & Trindade [58], who found that the four anthocyanins cyanidin-3-glucoside, cyanidin-3-rhamnoside, cyanidin-3-galactoside, and peonyin-3-O glucoside are present in black, purple, and red rice. Similarly, ultrasonic-assisted characterization of Thai rice berry bran by [20] identified anthocyanin 3-glucoside and paeoniflorin 3-glucoside as the main anthocyanins. As can be seen from Table 3, cyanidin-3-glucoside was the predominant anthocyanin, followed by cyanidin-3-galactoside and cyanidin-3- diglucoside. However, cyanidin-3-Rhamnoglucoside and malvidin-3-galactoside were quantified in trace amounts. Nevertheless, numerous studies have demonstrated that anthocyanin-3-O-glucoside and paeoniflorin-3-O-glucoside are the primary anthocyanins in black rice, accounting for 84% and 61% of TAC, respectively, depending on the color of the rice bran and the rice portion [2]. Yawadio & Morita [59] discovered two anthocyanins in black rice (cyanidin-3-glucoside and paeoniflorin-3-glucoside). A study on the effects of ultrasonic treatment on the content and bioaccessibility of anthocyanins in blueberries discovered that ultrasonic treatment greatly boosted the quantity of anthocyanins extracted from the berries and their bioaccessibility [60].

**Table 3:** Effect of ultrasound-assisted hot-air drying on selenium-enriched germinated black rice regarding phenolic profile, flavonols, and anthocyanins.

Compounds	RT (min)	Concentration range (µg. g^-1^dm)										
		Con1	Con2	Con3	US-SeGBR1	US-SeGBR2	US-SeGBR3	US-SeGBR4	US-SeGBR5	US-SeGBR6	US-SeGBR7	US-SeGBR8
*Phenolics*												
Caffeic acid	72.85	49.68c	47.36c	47.06c	48.88c	47.62c	47.81c	54.67b	49.01c	48.10c	74.95a	53.64b
Chlorogenic acid	12.04	41.135h	41.71h	44.74g	50.93de	49.02ef	46.98g	88.67b	60.283c	52.25d	151.62a	47.59fg
Ellagic acid	63.06	181.33gh	182.45gh	180.73h	189.26de	189.47d	186.46ef	247.99b	184fg	192.65c	499.69a	184.14fg
Ferulic acid	70.50	54.09a	54.37a	54.11a	53.48a	53.63a	53.92a	52.42a	53.80a	53.20a	37.02b	54.40a
Gallic acid	59.90	702.59h	946g	1255.45c	1222.78d	1137.90f	1205.61e	2605.94a	547.32i	1455.15b	396.4j	266.53k
Hydroxybenzoic acid	26.75	41.68b	22.44d	11.66f	3.81h	4.94gh	14.34e	63.44a	37.17c	6.58g	9.53f	3.50i
Isoferulic acid	71.76	289.33b	289.66ab	289.66ab	290.43ab	290.25ab	289.99ab	292.29a	291.43ab	289.69ab	289.43ab	289.54ab
*P*-coumaric acid	12.60	7.86b	8.20b	7.84b	7.90b	7.37b	7.83b	6.20b	6.50b	6.84b	13.22a	8.52b
Protocatechuic acid	13.07	246.71i	311.22g	418.59d	434.34c	384.10f	400.58e	908.42a	197.09j	469.79b	115.22k	252.84h
Sinapic acid	69.89	186.32a	186.74a	185.38a	183.89ab	181.51bc	180.24c	171.24e	183.99ab	176.38d	75.3	181.83bc
Vanillic acid	44.90	51.24b	46.53c	22.25g	6.55h	32.18e	37.51d	52.5b	67.57a	26.80f	31.87e	68.47a
Total phenolic acids	---	1851.96	2136.68f	2518.13c	2193.92e	2377.99e	2471.27d	4554.75a	1380.23j	2751.43b	1404.82i	1117.96k
*Flavonols*												
Kaempferol	70.34	48.42c	48.74c	48.76c	48.56c	48.83c	48.90c	51.22bc	52.116b	49.08c	62.39a	52.34b
Quercetin	69.90	38.75d	39.24d	39.15d	39.79cd	41.41cd	39.11d	42.32c	45.44b	39.5cd	48.18ab	49.88a
Rutin	12.09	3.87c	4.12c	4.21c	5.05bc	4.429c	4.26c	6.77bc	5.18bc	4.2c	11.51a	7.44b
Total Flavonols		91.04	92.10ij	92.12i	93.40f	92.77h	94.66e	100.31d	149.35a	92.78g	122.08b	109.66c
*Anthocyanins*	---											
Cyanidin-3-galactoside	13.25	359.825c	360.17bc	362.26b	360.14bc	366.69a	364.81a	361.47bc	361.46bc	360.61bc	N.D.	ND.
Cyanidin-3-glucoside	12.37	236.41i	241.74g	239.38gh	262.91e	239.4gh	237.5hi	380.69d	1965.77b	258.73f	2036.45a	906.28c
Cyanidin-3- diglucoside	13.02	200def	197.07fg	197.35fg	196.2g	198.92efg	198.85efg	205.95c	300.76a	209.95b	201.67de	202.89d
Cyanidin-3-Rhamnoglucoside	13.21	2.2a	2.57a	3.43a	2.84a	5.05a	5.13a	4.28a	3.13a	2.26a	4.22a	5.09a
Malvidin-3-galactoside	9.88	ND.	N.D.	200.65a	200.78a	202.51a	N.D.	201.79a	N.D.	N.D.	N.D.	N.D.
Cyanidin-3-gentiobioside	N.D.	ND.	ND.	ND.	ND.	ND.	ND.	ND.	ND.	ND.	ND.	ND.

Con = Control; US-SeGBR = Ultrasound selenium-enriched germinated black rice.

**Fig. 4:**
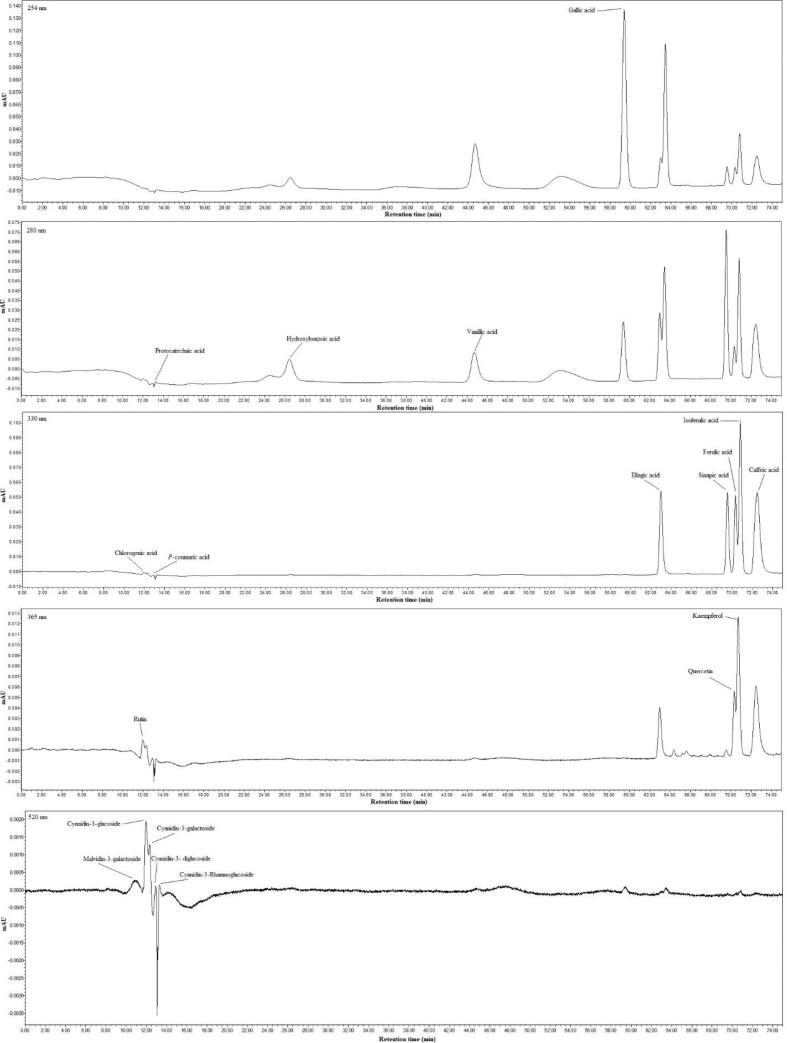
HPLC chromatograms on a different wavelength of phenolic and anthocyanins profile.

Spectral characteristics are precious for classifying anthocyanins, particularly for identifying their type. The HPLC chromatogram of SeGBR at 520 nm reveals multiple peaks (Fig. 4) that correspond to five anthocyanin compounds and distinct treatment groups. According to Lee [61], the average content of anthocyanin-3-glucoside was 45.52 mg/100 g, while paeoniflorin-3-glucoside was 3.22 mg/100 g. Hiemori et al. [62] determined that raw black rice contains 0.57 0.02 mg/g dw cyanidin-3-glucoside and 0.029 0.002 mg/g dw peonidin-3-glucoside. Various anthocyanins identified present in pigmented rice, such as cyanidin 3-glucoside, cyanidin 3-galactoside, cyanidin 3-rutinoside, cyanidin 3,5-diglucoside, malvidin 3-galactoside, peonidin 3-glucoside, and pelargonidin 3,5-diglucoside [61], [63]. They found that anthocyanin 3-glucoside was the most abundant form of anthocyanin in black and red rice, making up 88 and 67%, followed by peony 3-glucoside. The third most abundant anthocyanin found in two cereals is cyanide diglucoside [63].

### 3.9 Volatile profiling of Se-GBR

The flavor and aroma of rice significantly impact the sensory characteristics of cereals [64]. GC–MS analysis of 12 samples extracted using SPME fibers and analyzed using MassLynx software identified >50 volatile compounds, excluding those that were untraceable or impure (Table 4). A total of 55 volatiles were identified, including 10 classes such as 4 alcohols, 1 acid, 6 aldehydes, 12 alkane, 4 alkenes, 7 alkaloids, 4 ester, 9 ketones, 6 phenol, and 2 pyrazines. Among these compounds, aldehydes were quantitatively the major groups detected only in ultrasound-treated samples, whereas alkane, ketone, and phenol were the second significant groups detected in both control and ultrasound-treated samples [65].

**Table 4:** Effect of ultrasound-assisted hot-air drying on selenium-enriched germinated black rice regarding volatile compounds.

Groups	Compounds	RT (min)												
			Con1	Con2	Con3	US-SeGBR1	US-SeGBR2	US-SeGBR3	US-SeGBR4	US-SeGBR5	US-SeGBR6	US-SeGBR7	US-SeGBR8	US-SeGBR9
A	Alcohols													
1	1-Hexanol, 2-ethyl-	12.7	–	–	–	0.0080	–	–	–	–	–	–	–	–
2	2,3-Butanediol	2.78	–	–	0.1413	–	–	0.3243	0.3243	0.5431	0.1382	0.0009	–	0.0023
3	3-Heptanol, 6-methyl-	12.35	–	–	–	–	–	–	–	0.0176	–	–	–	–
4	2,4,7,9-Tetramethyl-5-decyn-4,7-diol	30.03	–	–	–	–	–	–	–	0.0052	0.0045	0.0007	–	–
B	Acids													
5	Butyric acid, 1-propylpentyl ester	25.4	–	–	–	–	–	–	–	–	–	0.0002	–	–
C	Aldehydes													
6	Benzeneacetaldehyde	13.08	–	–	–	0.0065	–	0.0078	0.0078	–	–	–	–	–
7	2-Octenal, (E)-	13.96	–	–	–	0.0042			–	–	–	–	–	–
–	–	–	–	–	–	–	–	–	–	–	–	–	–	–
J	Pyrazines													
54	Pyrazine, tetramethyl-	15.27	0.0024	–	–	0.0400	–	0.0672	0.0672	0.0784	0.0136	0.0006	0.0036	0.0071
55	2,3,5-Trimethyl-6-ethylpyrazine	18.99	–	–	–	0.0092	–	0.0177	0.0177	0.0087	–	–	–	–

*Complete table of volatile compounds is available in supplementary file; Volatile compounds are divided into 9 groups in alphabetical order form A to J; Con = Control; US-SeGBR = Ultrasound selenium-enriched germinated black rice; ‘-‘ notify not detected. Note: NIST11 (National Institute of Standards and Technology (NIST) Mass Spectral Library) database was used to identify compounds.

**Fig. 5:**
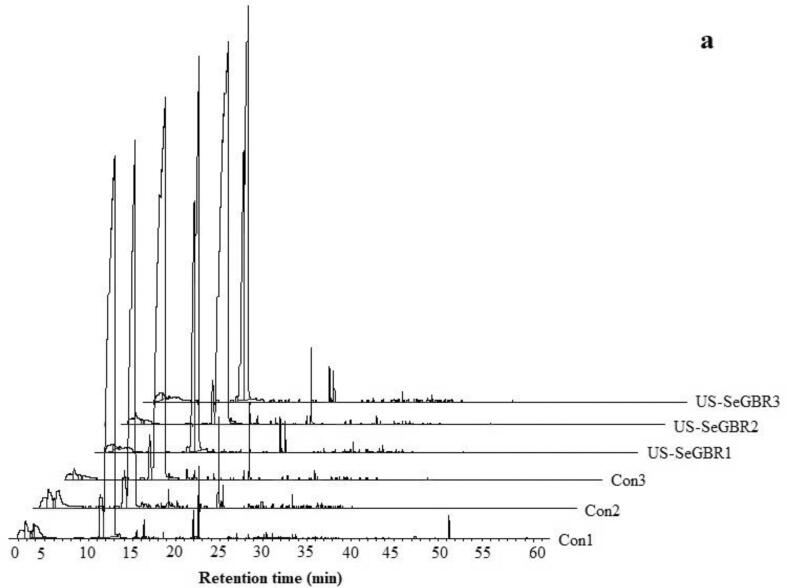

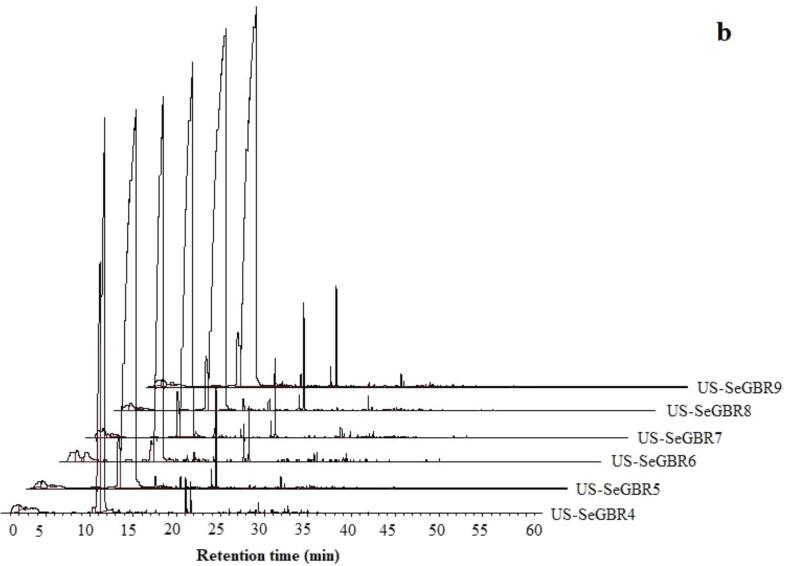
GC–MS peaks of volatile compounds of black rice.

The establishment of lipid secondary metabolites during linoleic acid degradation resulted in the formation of volatile aldehydes and alkanes via autooxidation and/or photosensitive oxidation of fatty acid esters hydroperoxide to form alkoxy radicals [6]. According to a study by [66], aldehydes and alkanes have been identified as crucial contributors to the aroma of rice due to their low odor threshold. These compounds are believed to be the primary source of barley and white wheat flour aroma. The results from the sample analysis could be used to determine the level of lipid oxidation and assess the quality of rice during storage. All of the ultrasound-treated samples contained alkanes dodecane and tetradecane. In addition, most compounds across 10 classes were identified in the ultrasound-treated samples compared to the control, indicating that ultrasound better preserves the volatile compounds at drying temperatures of 50, 60, and 70 °C. Similar research reportedly demonstrates that ultrasound pretreatment significantly impacts volatile components, aldehydes, and ketones, the essential aromatic compounds [67]. The proportion of aldehydes, alkanes, ketones, and phenols in every sample exceeded 75%. Compared to the untreated sample, the total content of compounds increased immediately after ultrasonic treatment for 10 min and 20 min, and the value increased as the ultrasonic time increased. The rise in compounds in ultrasonic SeGBR agrees with previous observations of germinated whole brown rice [68]. This is believed to result from the destruction of cell structures that leads to the release of free fatty acids (FFA) and the subsequent interactions between FFA and macromolecules. The variations observed in the total amount of identified volatile components may be linked to negative impacts related to lipid oxidation and FFA formation in response to various treatments [69]. However, there was a statistically significant difference (P > 0.05) in the drying of the samples after sonication. Despite being the most abundant classes, none of the acids are known to be associated with rice flavor. Fig. 5a & 5b show the results of a detailed analysis of the volatile components of samples subjected to varying degrees of ultrasound and drying, suggesting that the effects of these processes on the volatile composition were qualitatively similar but quantitatively different for specific components. According to Table 4, the compound most impacted by drying is butyric acid, 1-propyl pentyl ester in the acid, which is found in US-SeGBR7. This is primarily due to glucosinolates (GLS) and their breakdown products, such as isothiocyanates, organic cyanides, oxazolidine thione, and thiocyanates, which give the rice its characteristic odor and flavor [70]. Compared to untreated samples, the concentrations of alcohols, acids, aldehydes, ketones, and other compounds in untreated samples are substantially lower and much lower. According to the findings, the ultrasound process significantly impacted the volatile compounds of selenium-enriched black rice, and the most abundant butyric acid compound, 1-propyl pentyl ester in acids in black rice, was also the most affected compound. Untreated samples showed a decrease in volatiles due to biotransformation and diffusion during steeping, aligning with our previously reported results [68]. A study on the effects of ultrasonic treatment on the volatile compounds of roasted coffee beans discovered that ultrasonic treatment increased the concentration of several volatile compounds, including pyrazines and furans, while decreasing the levels of others, such as 2,3-pentanedione and 2-methyl pyrazine [71]. Another study on the effects of ultrasonic treatment on the volatile profile of Shanlan rice discovered that ultrasonic treatment resulted in significant changes in the concentration and composition of several volatile compounds, including an increase in the levels of ethyl hexanoate and a decrease in the levels of other volatile compounds like ethyl caprylate [72]. The results here reveal that the volatile compounds in dried black rice undergo distinct changes depending on the ultrasonic treatment and drying method. In order to better understand the effects of ultrasound and drying technology on the volatile compounds of selenium-rich germinated black rice, more research is needed to clarify the mechanism and pathway of volatiles derivation under techniques.

### 3.10 Microstructural changes

**Fig. 6:**
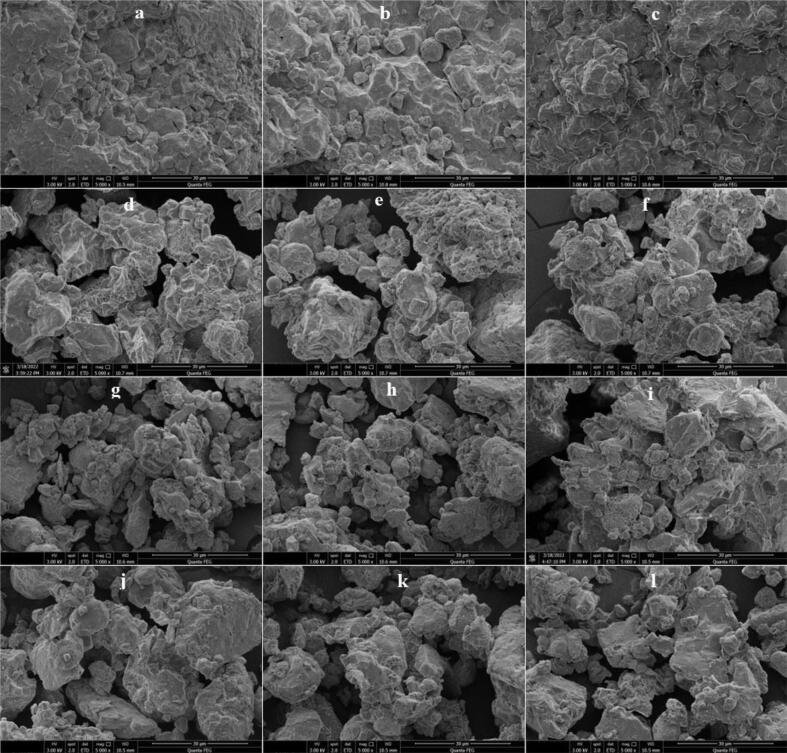
Effect of ultrasound and drying temperatures on the microstructure of SeGBR.

The microstructure of dried selenium-rich black rice, both untreated and after ultrasonic treatment, has been shown to change significantly (Fig. 6a−6l). The microstructure modification and starch gelatinization properties of ultrasonically-dried SeGBR were analyzed to understand better the factors that contributed to the observed changes in drying characteristics. Water diffusion and evaporation in SeGBR may be related to microstructure changes caused by ultrasound and hot-air drying. Fig. 6a-c demonstrates the compact structure and regular arrangement of starch particles in untreated SeGBR. Starch granules' tightly packed system is slightly broken up during thermal drying. According to a study by Shen et al. [73], in the initial drying stage, intense moisture diffusion within SeGBR caused the microscopic pores in the starch structure to collapse due to irradiation. This collapse facilitated the process of moisture diffusion and evaporation, leading to an increased drying rate. The surfaces of the samples that were sonicated for 10 min at 50, 60, and 70 °C exhibit gaps and deformations, notably along the cell walls of adjacent cells, as depicted in Fig. 6d–f. The surfaces of the rice samples sonicated for 20 min in Fig. 6g–i was nearly identical. The formation of these pores and cracks on the microscale (µm scale) is related to cavitation bubbles and results in microscale physical activities like shock waves, moisture jets impinging on solid–liquid interfaces at speeds of up to 200 m/s, and high-stress rates triggered by high-frequency heating/cooling rates [74]. According to reports, the collapse and cavitation bubbles that occur on or near the plant surface cause micro-cracks on the thin soybean flakes [5]. The alterations in surface structure would also increase mass transfer. Fig. 6j-1 shows that ultrasonic treatment of black rice for 50 min also altered its original form. There were also visible fissures on the outermost layer. These alterations in rice's microstructure may be explained by the cavitation effect produced by ultrasonic and enzymatic breakdown of cellulose. As seen in Fig. 1, Fig. 2, these effects improve water absorption during drying, reducing drying time.

The modification of the surface morphology also results in improved mass transfer. For instance, when ultrasound was applied to mung beans, hydration time was decreased by 25% Miano et al. [75], while it lowered hydration time in maize kernels by 35%. [76]. In the present research, the microscopic pores on the surface of germinated rice grains give a new path for water to enter the rice grains. As a result, ultrasonic pretreatment of SeGBR is predicted to improve hydration and minimize drying time. Furthermore, ultrasonic stimulation accelerated the pattern of morphological change, as evidenced by starch erosion over untreated samples with higher prominence and lower starch diameter. The findings indicated that ultrasonic pretreatment might increase starch granule disintegration during germination.

### 3.11 Effect of ultrasound and drying on Se content

**Fig. 7:**
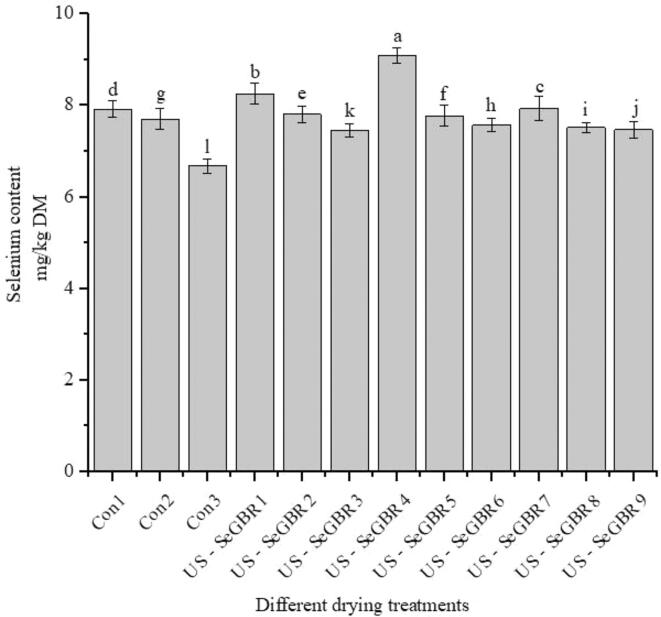
Effect of ultrasound and drying temperatures on the selenium content of SeGBR.

Significant differences (p < 0.05) were found in the selenium concentration of the germinated black rice samples (Fig. 7). Changes in the selenium concentration of various samples may result from the absorption of selenium solution throughout the 60-h germination period. Seeds absorb selenite to produce selenides through the catalysis of glutathione. When catalyzed by amino acid synthetases, Selenides generate selenium-containing amino acids, including SeCys and SeMet.

The selenium concentration of black rice was increased in US-SeGBR4 and US-SeGBR1 but decreased in Con3 at 70 °C. The findings are consistent with the previously reported range of brown rice selenium levels, 2.39 lg/100 g [4]. As indicated in Fig. 7, the selenium content of black rice varied considerably with various treatments (p < 0.05). A decrease in temperature was seen between the control sample and the ultrasonic treatment sample, which ranged in temperature from 50 °C to 70 °C. However, the value of selenium in the samples treated with ultrasound was somewhat more significant than in the control group. High levels of ultrasonic energy can increase the amount of organic selenium produced. This happens because ultrasound disrupts the tissue structure of plants, making it easier to extract phytochemicals and thereby raising the content of selenium [77]. An investigation into the effects of ultrasonic treatment on selenium bioavailability in wheat discovered that ultrasonic treatment significantly improved selenium bioavailability through promoting the release of bound selenium and altering it into more bioavailable forms [78]. Another investigation on the effects on selenium bioavailability in soybean sprouts discovered that selenium biofortification greatly enhanced total selenium content and bioavailability of selenium in the sprouts [79].

The organic selenium level of black rice was similarly impacted by drying temperature. Lower drying temperatures, such as 50 °C, have greater selenium concentration than 60 and 70 °C. When the drying temperatures were set to 50 °C, the selenium concentration of the Us-SeGBR4 (10 min) sonicated sample was 1.05 times greater than that of the control samples. Higher drying temperatures may inhibit amino acid synthase activity, resulting in less selenium generated during drying; hence, lower drying temperatures are more likely to increase selenium retention [3]. Reyes et al. [80] concluded that drying reduced broccoli's total selenium concentration by 35% compared to fresh selenium-rich veggies.

## 4 Conclusion

This study investigated the changes in the main quality features of ultrasonically treated selenium-enhanced germinated black rice throughout the hot-air drying process for the first time. In contrast to the control and US-SeGBR pretreatments, substantial differences in drying kinetics, moisture loss, selenium impact, and quality metrics were observed throughout the drying process of germinated black rice. Compared to traditional hot-air drying, the use of ultrasound in conjunction with hot air can result in a significant improvement in drying speed and a reduction in the drying time. The increased ultrasonic timings and drying temperatures enhanced the effectiveness. However, the ultrasound's strengthening effects were diminished by the decrease in the water content of SeGBR after drying. The ultrasound-treated group required a minimal activation energy of 3.97 kJ/mol to begin the drying process after 20 min, demonstrating the usefulness of ultrasound in the drying process. Although all fifteen models matched the experimental data sufficiently, the Hii model suited the drying kinetics of SeGBR the best, with the greatest R^2^ (>0.997 to 1.00), lowest RMSE (0.0027 to 0.0062), and lowest RSS (0.00) for all drying settings. It was discovered that phenolic profile and anthocyanins content might be raised with ultrasound-enhanced hot-air drying. The volatile compounds profile was significantly enhanced in the sonicated samples using HS-SPME and GC–MS analyses. The key benefit of using ultrasound in conjunction with hot-air drying for preserving the quality of selenium-enriched black garlic (SeGBR) is the preservation of organic selenium. SEM micrographs taken during the process confirmed the presence of micro cavitation in the grains, contributing to increased mass transfer. Based on these results, it can be concluded that integrating ultrasonic technology into the drying process can significantly speed it up while maintaining the quality of the dried black rice.

## CRediT authorship contribution statement

**Muhammad Tayyab Rashid:** Data curation, Methodology, Writing – original draft. **Kunlun Liu:** Conceptualization, Supervision. **Deng-Zhong Wei:** Software. **Mushtaque Ahmed Jatoi:** Writing – review & editing. **Qingyun Li:** Visualization. **Frederick Sarpong:** Visualization.

## Declaration of Competing Interest

The authors declare that they have no known competing financial interests or personal relationships that could have appeared to influence the work reported in this paper.

## References

[1] Jha P., Das A.J., Deka S.C. (2017). Optimization of ultrasound and microwave assisted extractions of polyphenols from black rice (*Oryza sativa* cv. Poireton) husk. J. Food Sci. Technol..

[2] Bolea C., Turturică M., Stănciuc N., Vizireanu C. (2016). Thermal degradation kinetics of bioactive compounds from black rice flour (*Oryza sativa* L.) extracts. J. Cereal Sci..

[3] Song Y., Tao Y., Zhu X., Han Y., Show P.L., Song C., Zaid H.F.M. (2019). Ultrasound-enhanced hot air drying of germinated highland barley seeds: drying characteristics, microstructure, and bioactive profile. AgriEngineering.

[4] Fang Y., Wang L., Xin Z., Zhao L., An X., Hu Q. (2008). Effect of foliar application of zinc, selenium, and iron fertilizers on nutrients concentration and yield of rice grain in China. J. Agric. Food Chem..

[5] Ding J., Hou G.G., Dong M., Xiong S., Zhao S., Feng H. (2018). Physicochemical properties of germinated dehulled rice flour and energy requirement in germination as affected by ultrasound treatment. Ultrason. Sonochem..

[6] Li Y., Liu K., Chen F. (2016). Effect of selenium enrichment on the quality of germinated brown rice during storage. Food Chem..

[7] Molan A.L. (2013). Antioxidant and prebiotic activities of selenium-containing green tea. Nutrition.

[8] Longchamp M., Castrec-Rouelle M., Biron P., Bariac T. (2015). Variations in the accumulation, localization and rate of metabolization of selenium in mature Zea mays plants supplied with selenite or selenate. Food Chem..

[9] Rashid M.T., Liu K., Jatoi M.A., Safdar B., Lv D., Li Q. (2022). Energy efficient drying technologies for sweet potatoes: operating and drying mechanism, quality-related attributes. Front. Nutr..

[10] Rashid M.T., Liu K., Han S., Jatoi M.A. (2022). The effects of thermal treatment on lipid oxidation, protein changes, and storage stabilization of rice bran. Foods.

[11] Rashid M.T., Ma H., Safdar B., Jatoi M.A., Wali A., Sarpong F., Zhou C. (2019). Synergy of ultrasound and osmotic dehydration in improving drying kinetics and quality of dried sweet potato (*Ipomea batatas* L.). J. Food Saf. Food Qual..

[12] Rashid M.T., Belščak-Cvitanović A., Karača S., Ma H., Komes D. (2019). Longan (*Dimocarpus longan*) and lychee (*Litchi chinensis*): functional ingredients in chocolate pralines. J. Food Biochem..

[13] Rashid M.T., Liu K., Hen S., Jatoi M.A., Sarpong F. (2023). Nutritional composition and volatile compounds stability in dry-heat and extruded stabilized rice bran during storage. Int. J. Food Sci. Technol..

[14] Tayyab Rashid M., Liu K., Ahmed Jatoi M., Safdar B., Lv D., Wei D. (2022). Developing ultrasound-assisted hot-air and infrared drying technology for sweet potatoes. Ultrason. Sonochem..

[15] Yang H., Gao J., Yang A., Chen H. (2015). The ultrasound-treated soybean seeds improve edibility and nutritional quality of soybean sprouts. Food Res. Int..

[16] Zhang G., Hu M., He L., Fu P., Wang L., Zhou J. (2013). Optimization of microwave-assisted enzymatic extraction of polyphenols from waste peanut shells and evaluation of its antioxidant and antibacterial activities in vitro. Food Bioprod. Process..

[17] Alimelli A., Pennazza G., Santonico M., Paolesse R., Filippini D., D’Amico A., Lundström I., Di Natale C. (2007). Fish freshness detection by a computer screen photoassisted based gas sensor array. Anal. Chim. Acta..

[18] Griglione A., Liberto E., Cordero C., Bressanello D., Cagliero C., Rubiolo P., Bicchi C., Sgorbini B. (2015). High-quality Italian rice cultivars: chemical indices of ageing and aroma quality. Food Chem..

[19] Liu K., Zhao S., Li Y., Chen F. (2018). Analysis of volatiles in brown rice, germinated brown rice, and selenised germinated brown rice during storage at different vacuum levels. J. Sci. Food Agric..

[20] Peanparkdee M., Yamauchi R., Iwamoto S. (2018). Characterization of Antioxidants extracted from thai riceberry bran using ultrasonic-assisted and conventional solvent extraction methods. Food Bioprocess Technol..

[21] Chungcharoen T., Prachayawarakorn S., Soponronnarit S., Tungtrakul P. (2012). Effect of drying temperature on drying characteristics and quality of germinated rices prepared from paddy and brown rice. Dry. Technol..

[22] Chemat F., Zill-e-Huma, Khan M.K. (2011). Applications of ultrasound in food technology: Processing, preservation and extraction. Ultrason. Sonochem..

[23] Saniso E., Prachayawarakorn S., Swasdisevi T., Soponronnarit S. (2020). Parboiled rice production without steaming by microwave-assisted hot air fluidized bed drying. Food Bioprod. Process..

[24] Santacatalina J.V., Rodríguez O., Simal S., Cárcel J.A., Mulet A., García-Pérez J.V. (2014). Ultrasonically enhanced low-temperature drying of apple: influence on drying kinetics and antioxidant potential. J. Food Eng..

[25] Wang Z., Sun J., Chen F., Liao X., Hu X. (2007). Mathematical modelling on thin layer microwave drying of apple pomace with and without hot air pre-drying. J. Food Eng..

[26] Cuccurullo G., Metallo A., Corona O., Cinquanta L. (2019). Comparing different processing methods in apple slice drying. Part 1. Performance of microwave, hot air and hybrid methods at constant temperatures. Biosyst. Eng..

[27] Xiao H.W., Le Pang C., Wang L.H., Bai J.W., Yang W.X., Gao Z.J. (2010). Drying kinetics and quality of Monukka seedless grapes dried in an air-impingement jet dryer. Biosyst. Eng..

[28] Dehghannya J., Hosseinlar S.H., Heshmati M.K. (2018). Multi-stage continuous and intermittent microwave drying of quince fruit coupled with osmotic dehydration and low temperature hot air drying. Innov. Food Sci. Emerg. Technol..

[29] Zhou Y., Cao S., Xi C., Li X., Zhang L., Wang G., Chen Z. (2020). A three dimension magnetic bio-char composite-based quick, easy, cheap, effective, rugged and safe method for multi-pesticides analysis of vegetables. J. Chromatogr. A.

[30] T.Q. Le, W. Jittanit, Drying kinetics of cooked jasmine brown rice during various drying methods, in: 22nd Natl. Grad. Res. Conf., Bangkok, Thailand, 2011, pp. 1–9.

[31] Tayyab Rashid M., Ahmed Jatoi M., Safdar B., Wali A., Muhammad Aadil R., Sarpong F., Ma H. (2020). Modeling the drying of ultrasound and glucose pretreated sweet potatoes: the impact on phytochemical and functional groups. Ultrason. Sonochem..

[32] Zhao L., Poh C.N., Wu J., Zhao X., He Y., Yang H. (2022). Effects of electrolysed water combined with ultrasound on inactivation kinetics and metabolite profiles of *Escherichia coli* biofilms on food contact surface. Innov. Food Sci. Emerg. Technol..

[33] Kaveh M., Amiri Chayjan R., Nikbakht A.M. (2017). Mass transfer characteristics of eggplant slices during length of continuous band dryer. Heat Mass Transf. Und Stoffuebertragung..

[34] Rashid M.T., Ma H., Jatoi M.A., Safdar B., El-Mesery H.S., Sarpong F., Ali Z., Wali A. (2019). Multi-frequency ultrasound and sequential infrared drying on drying kinetics, thermodynamic properties, and quality assessment of sweet potatoes. J. Food Process Eng..

[35] Sadaka G., Ubhi S., Atungulu G.S. (2016). Effects of initial moisture content and heating rate on wheat (oakes) drying kinetic parameters. Int. J. Eng. Sci. Res. Technol..

[36] de Oliveira G.H.H., Corrêa P.C., Araujo E.F., Valente D.S.M., Botelho F.M. (2010). Desorption isotherms and thermodynamic properties of sweet corn cultivars (*Zea mays* L.). Int. J. Food Sci. Technol..

[37] Sarpong F., Jiang H., Oteng-Darko P., Zhou C., Amenorfe L.P., Mustapha A.T., Rashid M.T. (2019). Mitigating effect of relative humidity (RH) on 2-furoylmethyl-Amino acid formation. Lwt.

[38] Miano A.C., Sabadoti V.D., Augusto P.E.D. (2018). Enhancing the hydration process of common beans by ultrasound and high temperatures: impact on cooking and thermodynamic properties. J. Food Eng..

[39] Nadi F., Tzempelikos D. (2018). Vacuum drying of apples (cv. Golden Delicious): drying characteristics, thermodynamic properties, and mass transfer parameters. Heat Mass Transf. Und Stoffuebertragung..

[40] Al-Zubaidy M.M.I., Khalil R.A. (2007). Kinetic and prediction studies of ascorbic acid degradation in normal and concentrate local lemon juice during storage. Food Chem..

[41] Goneli A.L.D., Corrêa P.C., de Oliveira G.H.H., De Oliveira A.P.L.R., Orlando R.C. (2016). Moisture sorption isotherms of castor beans. Part 2: thermodynamic properties. Rev. Bras. Eng. Agric. e Ambient..

[42] Balbinoti T.C.V., Jorge L.M.d.M., Jorge R.M.M. (2018). Modeling the hydration step of the rice (Oryza sativa) parboiling process. J. Food Eng..

[43] Jideani V.A., Mpotokwana S.M. (2009). Modeling of water absorption of Botswana bambara varieties using Peleg’s equation. J. Food Eng..

[44] Borges C.W.C., Jorge L.M.d.M., Jorge R.M.M. (2017). Kinetic modeling and thermodynamic properties of soybean cultivar (BRS257) during hydration process. J. Food Process. Eng..

[45] Souza J.L.F., Oliveira D.E.C., Plácido G.R., Egea M.B., Caliari M., da Silva M.A.P. (2019). Thermodynamic and nutritional properties and drying kinetics of pequi (Caryocar brasiliense cambess) mesocarp. Rev. Bras. Eng. Agric. e Ambient..

[46] Mihindukulasuriya S.D.F., Jayasuriya H.P.W. (2015). Drying of chilli in a combined infrared and hot air rotary dryer. J. Food Sci. Technol..

[47] Bantle M., Eikevik T.M. (2014). A study of the energy efficiency of convective drying systems assisted by ultrasound in the production of clipfish. J. Clean. Prod..

[48] Abdoli B., Zare D., Jafari A., Chen G. (2018). Evaluation of the air-borne ultrasound on fluidized bed drying of shelled corn: effectiveness, grain quality, and energy consumption. Dry. Technol..

[49] Dibagar N., Amiri Chayjan R. (2019). Rough rice convective drying enhancement by intervention of airborne ultrasound–a response surface strategy for experimental design and optimization. Dry. Technol..

[50] Ruangchakpet A., Sajjaanantakul T. (2007). Effect of Browning on Total Phenolic, Flavonoid Content and Antioxidant Activity in Indian Gooseberry (*Phyllanthus emblica* Linn.). Nat. Sci..

[51] Hrnčič M.K., Cör D., Kotnik P., Knez Ž. (2019). Extracts of white and red grape skin and rosehip fruit: Phenolic compounds and their antioxidative activity. Acta Chim. Slov..

[52] Aguilar-Camacho M., Welti-Chanes J., Jacobo-Velázquez D.A. (2019). Combined effect of ultrasound treatment and exogenous phytohormones on the accumulation of bioactive compounds in broccoli florets. Ultrason. Sonochem..

[53] Talcott S.T., Duncan C.E., Del Pozo-Insfran D., Gorbet D.W. (2005). Polyphenolic and antioxidant changes during storage of normal, mid, and high oleic acid peanuts. Food Chem..

[54] Chen L., Wu J., Li Z., Liu Q., Zhao X., Yang H. (2019). Metabolomic analysis of energy regulated germination and sprouting of organic mung bean (*Vigna radiata*) using NMR spectroscopy. Food Chem..

[55] Zaro M.J., Ortiz L.C., Keunchkarian S., Chaves A.R., Vicente A.R., Concellón A. (2015). Analía Concellón, chlorogenic acid retention in white and purple eggplant after processing and cooking. Lwt.

[56] Qiu Y., Liu Q., Beta T. (2010). Antioxidant properties of commercial wild rice and analysis of soluble and insoluble phenolic acids. Food Chem..

[57] Zeng J., Dou Y., Yan N., Li N.a., Zhang H., Tan J.-N. (2019). Optimizing ultrasound-assisted deep eutectic solvent extraction of bioactive compounds from Chinese wild rice. Molecules.

[58] Goufo P., Trindade H. (2014). Rice antioxidants: phenolic acids, flavonoids, anthocyanins, proanthocyanidins, tocopherols, tocotrienols, c-oryzanol, and phytic acid. Food Sci. Nutr..

[59] Yawadio R., Morita N. (2007). Color enhancing effect of carboxylic acids on anthocyanins. Food Chem..

[60] García-Salas P., Gómez-Caravaca A.M., Arráez-Román D., Segura-Carretero A., Guerra-Hernández E., García-Villanova B., Fernández-Gutiérrez A. (2013). Influence of technological processes on phenolic compounds, organic acids, furanic derivatives, and antioxidant activity of whole-lemon powder. Food Chem..

[61] Lee J.H. (2010). Identification and quantification of anthocyanins from the grains of black rice (*Oryza sativa* L.) varieties. Food Sci. Biotechnol..

[62] Hiemori M., Koh E., Mitchell A.E. (2009). Influence of cooking on anthocyanins in black rice (*Oryza sativa* L. japonica var. SBR). J. Agric. Food Chem..

[63] Abdel-Aal E.S.M., Young J.C., Rabalski I. (2006). Anthocyanin composition in black, blue, pink, purple, and red cereal grains. J. Agric. Food Chem..

[64] Xia Q., Li Y. (2018). Ultra-high pressure effects on color, volatile organic compounds and antioxidants of wholegrain brown rice (*Oryza sativa* L.) during storage: a comparative study with high-intensity ultrasound and germination pretreatments. Innov. Food Sci. Emerg. Technol..

[65] Condurso C., Cincotta F., Tripodi G., Verzera A. (2017). Bioactive volatiles in Sicilian (South Italy) saffron: safranal and its related compounds. J. Essent. Oil Res..

[66] Cramer A.C.J., Mattinson D.S., Fellman J.K., Baik B.K. (2005). Analysis of volatile compounds from various types of barley cultivars. J. Agric. Food Chem..

[67] Pei Y., Li Z., Xu W., Song C., Li J., Song F. (2021). Effects of ultrasound pretreatment followed by far-infrared drying on physicochemical properties, antioxidant activity and aroma compounds of saffron (*Crocus sativus* L.). Food Biosci..

[68] Xia Q., Wang L., Xu C., Mei J., Li Y. (2017). Effects of germination and high hydrostatic pressure processing on mineral elements, amino acids and antioxidants in vitro bioaccessibility, as well as starch digestibility in brown rice (*Oryza sativa* L.). Food Chem..

[69] Xia Q., Wang L., Yu W., Li Y. (2017). Investigating the influence of selected texture-improved pretreatment techniques on storage stability of wholegrain brown rice: involvement of processing-induced mineral changes with lipid degradation. Food Res. Int..

[70] Xu B.G., Zhang M., Bhandari B., Cheng X.F., Islam M.N. (2015). Effect of ultrasound-assisted freezing on the physico-chemical properties and volatile compounds of red radish. Ultrason. Sonochem..

[71] Zhai X., Yang M., Zhang J., Zhang L., Tian Y., Li C., Bao L., Ma C., Abd El-Aty A.M. (2022). Feasibility of ultrasound-assisted extraction for accelerated cold brew coffee processing: characterization and comparison with conventional brewing methods. Front. Nutr..

[72] Yang Z., Lin X., Wang L.u., Li C., Liu S. (2020). Effects of ultrasonic treatment on the cooking and fermentation properties of Shanlan rice. J. Cereal Sci..

[73] Shen L., Gao M., Zhu Y., Liu C., Wang L., Kamruzzaman M.d., Liu C., Zheng X. (2021). Microwave drying of germinated brown rice: correlation of drying characteristics with the final quality. Innov. Food Sci. Emerg. Technol..

[74] Kentish S., Feng H. (2014). Applications of power ultrasound in food processing. Annu. Rev. Food Sci. Technol..

[75] Miano A.C., Pereira J.D.C., Castanha N., Júnior M.D.D.M., Augusto P.E.D. (2016). Enhancing mung bean hydration using the ultrasound technology: description of mechanisms and impact on its germination and main components. Sci. Rep..

[76] Miano A.C., Ibarz A., Augusto P.E.D. (2017). Ultrasound technology enhances the hydration of corn kernels without affecting their starch properties. J. Food Eng..

[77] Ren F., Perussello C.A., Zhang Z., Kerry J.P., Tiwari B.K. (2018). Impact of ultrasound and blanching on functional properties of hot-air dried and freeze dried onions. Lwt.

[78] Trakselyte-Rupsiene K., Juodeikiene G., Cernauskas D., Bartkiene E., Klupsaite D., Zadeike D., Bendoraitiene J., Damasius J., Ignatavicius J., Sikorskaite-Gudziuniene S. (2021). Integration of ultrasound into the development of plant-based protein hydrolysate and its bio-stimulatory effect for growth of wheat grain seedlings in vivo. Plants.

[79] Huang Y., Lei N., Xiong Y., Liu Y., Tong L., Wang F., Fan B., Maesen P., Blecker C. (2022). Influence of selenium biofortification of soybeans on speciation and transformation during seed germination and sprouts quality. Foods.

[80] Reyes A., Mahn A., Guzmán C., Antoniz D. (2012). Analysis of the drying of broccoli florets in a fluidized pulsed bed. Dry. Technol..

