# Supplementary material for: Drying kinetics and quality dynamics of ultrasound-assisted dried selenium-enriched germinated black rice
Source: Ultrason Sonochem. 2023 Jun 7;98:106468. doi: 10.1016/j.ultsonch.2023.106468 (PMC10422111; doi:10.1016/j.ultsonch.2023.106468)
Supplement: Supplementary data 1 [file mmc1.docx]

**Supplementary data**

Supplementary Table S1. Drying models used to study drying kinetic of black rice

| **S. No** | **Model name** | **Model** | **References** |
| --- | --- | --- | --- |
| 1 | Newton | $Mr=exp(-kt)$ | [1] |
| 2 | Page | $Mr=exp(-kt^{n}$) | [2] |
| 3 | Modified Page 1 | $Mr=aexp(-kt^{n}$) | [3] |
| 4 | Handerson and Pabis model | $Mr=aexp(-kt^{n}$) | [4] |
| 5 | Modified Henderson and Pabis | $Mr=aexp\left( -kt \right)+bexp\left( -gt \right)+cexp(-ht)$ | [5] |
| 6 | Logarithmic model | $Mr=aexp\left( -kt \right)+c$ | [6] |
| 7 | Midilli model | $Mr=aexp\left( -kt \right)+bt$ | [7] |
| 8 | Two-term model | $Mr=aexp\left( {-k}_{1}t \right)+bexp\left( {-k}_{2}t \right)$ | [8] |
| 9 | Two-term exponential | $Mr=aexp\left( -kt \right)+\left( 1-a \right)exp\left( -kat \right)$ | [9] |
| 10 | Hii model | $Mr=aexp\left( {-k}_{1}t^{n} \right)+bexp\left( {-k}_{2}t^{n} \right)$ | [10] |
| 11 | Verma model | $Mr=aexp\left( -kt \right)+\left( 1-a \right)exp(-gt)$ | [11] |
| 12 | Modified Midilli model | $Mr=aexp\left( -kt \right)+b$ | [12] |
| 13 | Aghbashlo model | $Mr=exp\left( k_{1}t/1+k_{2}t \right)$ | [13] |
| 14 | Wang and Singh | $Mr=1+at+bt^{2}$ | [14] |
| 15 | Silva model | $Mr=exp(-at-b\sqrt{t})$ | [15] |

Supplementary Table S2. Averages of selected models fitted to drying using the US and HAD for germinated black rice

| **Sample Codes** | **T (^°^C)** | **Coefficients** | **R^2^** | **RSS** | **χ^2^** | **RMSE** |
| --- | --- | --- | --- | --- | --- | --- |
| **Modified Henderson and Pabis model** | | | | | | |
| Con1  Con2  Con3  US-SeGBR1  US-SeGBR2  US-SeGBR3  US-SeGBR4  US-SeGBR5  US-SeGBR6  US-SeGBR7  US-SeGBR8  US-SeGBR9 | 50  60  70  50  60  70  50  60  70  50  60  70 | a.0.602, k.0.003, b.0.005, g.-0.012, c.0.392, h. 0.082  a.0.457, k.0.001, b.0.107, g.0.130, c.0.433, h. 0.033  a.0.556, k.0.034, b.0.570, g.-0.002, c.-0.159, h. -0.006  a.0.478, k.0.001, b.0.143, g.0.379, c.0.379, h. 0.028  a.0.369, k.0.064, b.0.617, g.0.004, c.0.000, h. -0.003  a.0.458, k.0.002, b.0.350, g.0.030, c.0.188, h. 0.109  a.0.692, k.0.007, b.0.056, g.-0.008, c.0.249, h. 0.158  a.0.634, k.0.004, b.0.353, g.0.060, c.0.000, h. -0.032  a.0.099, k.-0.007, b.0.666, g.0.012, c.0.214, h. 0.144  a.0.357, k.0.082, b.0.627, g.0.003, c.0.000, h. -0.033  a.0.676, k.0.005, b.0.000, g.-0.032, c.0.317, h. 0.090  a.0.585, k.0.015, b.0.197, g.-0.004, c.0.218, h. 0.125 | 0.999  0.996  0.987  0.999  0.995  0.998  0.999  0.995  0.999  0.989  0.996  0.997 | 0.00  0.00  0.01  0.00  0.00  0.00  0.00  0.00  0.00  0.00  0.00  0.00 | 1.36×10^-05^  0.00017 0.00105  4.43×10^-04^  0.00026 4.15×10^-05^  5.27×10^-05^  0.00024 3.37×10^-05^  0.00043 0.00012  4.31×10^-05^ | 0.00244  0.00835  0.01922  0.01105  0.00616  0.01057  0.00295  0.00493  0.00296  0.01427  0.00855  0.00324 |
| **Midilli model** | | | | | | |
| Con1  Con2  Con3  US-SeGBR1  US-SeGBR2  US-SeGBR3  US-SeGBR4  US-SeGBR5  US-SeGBR6  US-SeGBR7  US-SeGBR8  US-SeGBR9 | 50  60  70  50  60  70  50  60  70  50  60  70 | a.0.651, k.0.043, n.0.011, b. -0.001  a.0.675, k.0.044, n.0.043, b. -0.002  a.0.001, k.-6.802, n.-0.035, b. -0.001  a.0.001, k.-7.541, n.-0.29, b. 0.000  a.0.000, k.-8.571, n.-0.030, b. 0.000  a.0.001, k.-7.341, n.-0.035, b. -0.001  a.0.699, k.0.035, n.0.012, b. -0.002  a.0.000, k.-8.871, n.-0.023, b. 0.000  a.0.000, k.-8.991, n.-0.030, b. 0.000  a.0.651, k.0.034, n.0.0981, b. -0.011  a.0.000, k.-7.861, n. -0.028, b. 0.000  a.0.000, k.-8.009, n.-0.034, b. 0.000 | 0.907  0.903  0.979  0.988  0.983  0.990  0.830  0.979  0.982  0.892  0.985  0.987 | 0.03  0.03  0.00  0.00  0.00  0.02  0.00  0.00  0.00  0.02  0.00  0.00 | 0.00266  0.00335  0.00033  0.00016 0.00036  8.47×10^-05^  0.00212 0.00035  0.00044 0.00181  0.00028 0.00020 | 0.03011  0.03885  0.01088  0.00960 0.08584  0.00894 0.03092  0.01332 0.01057  0.03011 0.01256  0.00945 |
| **Two term model** | | | | | | |
|  | | | | | | |
| Con1  Con2  Con3  US-SeGBR1  US-SeGBR2  US-SeGBR3  US-SeGBR4  US-SeGBR5  US-SeGBR6  US-SeGBR7  US-SeGBR8  US-SeGBR9 | 50  60  70  50  60  70  50  60  70  50  60  70 | a.0.440, k1.0.065, b.0.553, k2. 0.002  a.0.475, k1.0.046, b.0.506, k2. 0.002  a.0.429, k1.0.050, b.0.551, k2. 0.003  a.0.428, k1.0.053, b.0.555, k2. 0.002  a.0.459, k1.0.048, b.0.524, k2. 0.002  a.0.413, k1.0.064, b.0.581, k2. 0.004  a.0.372, k1.0.067, b.0.615, k2. 0.002  a.0.443, k1.0.044, b.0.537, k2. 0.002  a.0.469, k1.0.048, b.0.515, k2. 0.002  a.0.398, k1.0.070, b.0.585, k2. 0.002  a.0.422, k1.0.053, b.0.550, k2. 0.003  a.0.448, k1.0.056, b.0.544, k2.0.003 | 0.995  0.993  0.989  0.992  0.993  0.996  0.989  0.992  0.993  0.982  0.990  0.995 | 0.24  0.26  0.26  0.24  0.27  0.27  0.21  0.25  0.26  0.23  0.26  0.27 | 0.02223  0.02933  0.04371  0.02180  0.03792  0.02555  0.02379  0.02785  0.06598  0.02075  0.02576  0.04485 | 0.10095  0.11489  0.12420  0.10951  0.12495  0.15305  0.10347  0.11802  0.12843  0.10186  0.11964  0.04485 |
| **Henderson and Pabis model** | | | | | | |
|  | | | | | | |
| Con1  Con2  Con3  US-SeGBR1  US-SeGBR2  US-SeGBR3  US-SeGBR4  US-SeGBR5  US-SeGBR6  US-SeGBR7  US-SeGBR8  US-SeGBR9 | 50  60  70  50  60  70  50  60  70  50  60  70 | k.0.166, n.0.329, a. 1.007  k.0.132, n.0.399, a. 1.008  k.0.107, n.0.453, a. 1.004  k.0.131, n.0.385, a. 1.006  k.0.116, n.0.431, a. 1.006  k.0.099, n.0.487, a. 1.004  k.0.122, n. 0.386, a. 1.003  k.0.107, n.0.438, a. 1.007  k.0.114, n.0.440, a. 1.007  k.0.149, n.0.354, a. 1.003  k.0.114, n.0.431, a. 1.005  k.0.112, n.0.448, a. 1.008 | 0.988  0.986  0.992  0.990  0.989  0.994  0.995  0.989  0.988  0.993  0.992  0.988 | 0.00  0.00  0.00  0.00  0.00  0.00  0.00  0.00  0.00  0.00  0.00  0.00 | 0.00032  0.00042  0.00018  0.00033  0.00034  0.00010  9.35×10^-05^  0.00032  0.00019  0.00011  0.00018  0.00042 | 0.01220  0.01374  0.00807  0.01354  0.01196  0.00970  0.00648  0.01266  0.00695  0.00753  0.01016  0.01343 |
|  |  |  |  |  |  |  |
| **Logarithmic model** | | | | | | |
|  | | | | | | |
| Con1  Con2  Con3  US-SeGBR1  US-SeGBR2  US-SeGBR3  US-SeGBR4  US-SeGBR5  US-SeGBR6  US-SeGBR7  US-SeGBR8  US-SeGBR9 | 50  60  70  50  60  70  50  60  70  50  60  70 | a.0.532, k.0.032, c. 0.419  a.0.573, k.0.031, c. 0.387  a.0.580, k.0.029, c. 0.377  a.0.539, k.0.030, c. 0.416  a.0.572, k.0.032, c. 0.393  a.0.578, k.0.038, c. 0.401  a.0.512, k.0.028, c. 0.431  a.0.561, k.0.029, c. 0.400  a.0.575, k.0.033, c. 0.395  a.0.520, k. 0.028, c. 0.412  a.0.567, k.0.029, c. 0.387  a.0.570, k.0.037, c. 0.408 | 0.968  0.987  0.984  0.981  0.988  0.992  0.972  0.987  0.990  0.962  0.982  0.990 | 0.01  0.01  0.01  0.01  0.01  0.00  0.01  0.01  0.00  0.01  0.01  0.00 | 0.00097  0.00068  0.00111  0.00060  0.00071  0.00025  0.00124  0.00061  0.00094  0.00136  0.00078  0.00032 | 0.02110  0.01750  0.01981  0.01830  0.01721  0.01541  0.02364  0.01758  0.01540  0.02610  0.02085  0.01186 |
|  |  |  |  |  |  |  |
| **Verma model (Non-Significant)** | | | | | | |
|  |  |  |  |  |  |  |
| Con1  Con2  Con3  US-SeGBR1  US-SeGBR2  US-SeGBR3  US-SeGBR4  US-SeGBR5  US-SeGBR6  US-SeGBR7  US-SeGBR8  US-SeGBR9 | 50  60  70  50  60  70  50  60  70  50  60  70 | a.-0.345, k.0.006, g. 0.006  a.-0.365, k.0.008, g. 0.008  a.-0.357, k.0.009, g. 0.009  a.-0.331, k.0.007, g. 0.007  a.-0.347, k.0.009, g. 0.009  a.-0.317, k.0.012, g. 0.012  a.-0.337, k.0.003, g. 0.003  a.-0.367, k.0.005, g. 0.005  a.-0.329, k.0.004, g.0.004  a.-0.369, k.0.002, g. 0.002  a.-0.349, k.0.003, g. 0.003  a.-0.375, k.0.007, g. 0.007 | 0.425  0.523  0.254  0.537  0.532  0.423  0.275  0.645  0.239  0.387  0.547  0.448 | 0.24  0.13  0.34  0.23  0.65  0.23  0.65  0.12  0.54  0.65  0.45  0.13 | 0.02490  0.01849  0.01648  0.01582  0.01636  0.00521  0.04234  0.00123  0.00543  0.00234  0.00764  0.00324 | 0.10683  0.09122  0.07628  0.09329  0.08208  0.06911  0.02343  0.03543  0.46456  0.53348  0.03454  0.34543 |
|  |  |  |  |  |  |  |
| **Silva Model** | | | | | | |
|  | | | | | | |
| Con1  Con2  Con3  US-SeGBR1  US-SeGBR2  US-SeGBR3  US-SeGBR4  US-SeGBR5  US-SeGBR6  US-SeGBR7  US-SeGBR8  US-SeGBR9 | 50  60  70  50  60  70  50  60  70  50  60  70 | a.-0.003, b. 0.108  a.-0.002, b. 0.107  a.-0.001, b. 0.098  a.-0.002, b. 0.101  a.-0.002, b. 0.102  a.-0.001, b. 0.098  a.-0.002, b. 0.094  a.-0.001, b. 0.095  a.-0.002, b. 0.102  a.-0.003, b. 0.104  a.-0.002, b. 0.100  a.-0.002, b. 0.103 | 0.997  0.993  0.993  0.996  0.992  0.995  0.998  0.992  0.991  0.997  0.995  0.990 | 0.00  0.00  0.00  0.00  0.00  0.00  0.00  0.00  0.00  0.00  0.00  0.00 | 0.00012  0.00020  0.00017  0.00013  0.00022  8.14×10^-05^  4.17×10^-05^  0.00028  0.00029  0.00010  0.00015  0.00046 | 0.00763  0.00967  0.00785  0.00861  0.00961  0.00863  0.00433  0.01186  0.00862  0.00708  0.00931  0.01418 |
|  |  |  |  |  |  |  |
| **Wang & Singh Model (Non-Significant)** | | | | | | |
|  | | | | | | |
| Con1  Con2  Con3  US-SeGBR1  US-SeGBR2  US-SeGBR3  US-SeGBR4  US-SeGBR5  US-SeGBR6  US-SeGBR7  US-SeGBR8  US-SeGBR9 | 50  60  70  50  60  70  50  60  70  50  60  70 | a.-0.008, b. 0.043  a.-0.010, b. 0.034  a.-0.011, b. 0.069  a.-0.010, b. 0.098  a.-0.011, b. 0.031  a.-0.034, b. 0.043  a.-0.031, b. 0.042  a.-0.010, b. 0.043  a.-0.023., b. 0.069  a.-0.009, b. 0.043  a.-0.011, b. 0.065  a.-0.013, b. 0.043 | 0.597  0.786  0.453  0.774  0.858  0.924  0.772  0.865  0.877  0.686  0.844  0.891 | 0.34  0.12  0.55  0.56  0.23  0.78  0.34  0.45  0.76  0.23  0.11  0.56 | 0.05435  0.00343  0.00233  0.02334  0.06456  0.02343  0.01234  0.04564  0.09894  0.06456  0.07533  0.08345 | 0.00253  0.04536  0.01345  0.04456  0.03456  0.06234  0.05234  0.08534  0.08654  0.09123  0.01236  0.00653 |
|  |  |  |  |  |  |  |
| **Aghbashlo Model (Non-Significant)** | | | | | | |
|  | | | | | | |
| Con1  Con2  Con3  US-SeGBR1  US-SeGBR2  US-SeGBR3  US-SeGBR4  US-SeGBR5  US-SeGBR6  US-SeGBR7  US-SeGBR8  US-SeGBR9 | 50  60  70  50  60  70  50  60  70  50  60  70 | K1.-0.326, k2. 0.319  K1.-0.305, k2. 0.296  K1.0.910, k2. -0.919  K1.0.708, k2. -0.715  K1.0.863, k2. -0.872  K1.8.715, k2. -8.727  K1.0.743, k2. -5.324  K1.-0.436, k2. 0.420  K1.-0.424, k2. 0.418  K1.7.432, k2. -0.987  K1.6.424, k2. -0.843  K1.0.410, k2. -0.453 | 0.117  0.468  0.623  0.417  0.592  0.754  0.535  0.234  0.723  0.498  0.312  0.642 | 0.45  0.12  0.34  0.10  0.45  0.54  0.23  0.65  0.65  0.24  0.56  0.34 | 0.05435  0.02435  0.02235  0.03538  0.09345  0.02134  0.05643  0.01234  0.06353  0.09823  0.02432  0.01345 | 0.05345  0.01232  0.04353  0.03455  0.06456  0.02432  0.06432  0.09123  0.03435  0.06575  0.05345  0.02342 |
|  |  |  |  |  |  |  |
| **Thompson model (Non-Significant)** | | | | | | |
|  | | | | | | |
| Con1  Con2  Con3  US-SeGBR1  US-SeGBR2  US-SeGBR3  US-SeGBR4  US-SeGBR5  US-SeGBR6  US-SeGBR7  US-SeGBR8  US-SeGBR9 | 50  60  70  50  60  70  50  60  70  50  60  70 | a.180.168, b. 424.555  a. 92.329, b. 262.961  a.40.969, b. 185.374  a.51.961, b. 201.555  a.58.886, b. 214.351  a.19.719, b. 139.722  a.93.955, b. 326.955  a.57.307, b. 232.147  a.50.749, b. 199.237  a.123.861, b. 344.822  a.54.726, b. 213.905  a.40.131, b. 180.662 | 0.962  0.945  0.966  0.950  0.955  0.981  0.974  0.957  0.950  0.958  0.963  0.957 | 21696.57  23615.71  8199.117  47583.23  15612.13  5516.795  31476.78  33389.68  1892.455  32222.18  45165.82  10946.58 | 1972.415  2623.967  1366.518  4325.748  2230.304  501.5265  3497.419  3709.964  473.1118  2929.289  4516.581  1824.429 | 30.066  34.362  21.961  48.776  30.304  21.441  39.671  43.069  10.875  38.270  50.092  27.962 |
|  |  |  |  |  |  |  |
| **Page model** | | | | | | |
|  | | | | | | |
| Con1  Con2  Con3  US-SeGBR1  US-SeGBR2  US-SeGBR3  US-SeGBR4  US-SeGBR5  US-SeGBR6  US-SeGBR7  US-SeGBR8  US-SeGBR9 | 50  60  70  50  60  70  50  60  70  50  60  70 | k.0.162, n. 0.332  k.0.128, n. 0.404  k.0.105, n. 0.456  k.0.127, n. 0.388  k.0.113, n. 0.435  k.0.098, n. 0.490  k.0.121, n. 0.388  k.0.104, n. 0.442  k.0.111, n. 0.444  k.0.147, n. 0.355  k.0.112, n. 0.434  k.0.109, n. 0.452 | 0.988  0.986  0.992  0.990  0.989  0.994  0.995  0.989  0.988  0.993  0.992  0.989 | 0.00  0.00  0.00  0.00  0.00  0.00  0.00  0.00  0.00  0.00  0.00  0.00 | 0.00031  0.00040  0.00018  0.00032  0.00039  0.00010  0.00011  0.00030  0.00016  0.00011  0.00018  0.00050 | 0.01203  0.01355  0.00807  0.01336  0.01270  0.00970  0.00719  0.01243  0.00649  0.00753  0.01016  0.01465 |
|  |  |  |  |  |  |  |
| **Newton (Non-Significant)** | | | | | | |
|  | | | | | | |
| Con1  Con2  Con3  US-SeGBR1  US-SeGBR2  US-SeGBR3  US-SeGBR4  US-SeGBR5  US-SeGBR6  US-SeGBR7  US-SeGBR8  US-SeGBR9 | 50  60  70  50  60  70  50  60  70  50  60  70 | k. 0.006  k. 0.008  k. 0.009  k. 0.007  k. 0.006  k. 0.005  k. 0.007  k. 0.004  k. 0.006  k. 0.004  k. 0.008  k. 0.005 | 0.117  0.458  0.645  0.417  0.534  0.633  0.243  0.634  0.354  0.452  0.165  0.643 | 0.12  0.43  0.23  0.43  0.15  0.18  0.12  0.45  0.54  0.23  0.25  0.19 | 0.04234  0.01453  0.05433  0.02433  0.05234  0.02134  0.09872  0.03424  0.05433  0.09872  0.02342  0.06545 | 0.00322  0.02343  0.04353  0.06546  0.09842  0.03254  0.02345  0.05435  0.02432  0.07674  0.05343  0.04234 |

Con= Control; US-SeGBR= Ultrasound selenium germinated enriched black rice

Supplementary Table S3.: Effect of US pretreatments and drying temperatures on germinated black rice regarding volatile compounds.

|  |  |  | Control | | | Ultrasound treated samples | | | | | | | | |
| --- | --- | --- | --- | --- | --- | --- | --- | --- | --- | --- | --- | --- | --- | --- |
| **No.** | **Compounds** | RT | Con1 | Con2 | Con3 | US-SeGBR1 | US-SeGBR2 | US-SeGBR3 | US-SeGBR4 | US-SeGBR5 | US-SeGBR6 | US-SeGBR7 | US-SeGBR8 | US-SeGBR9 |
|  |  |  | 50^o^C | 60^o^C | 70^o^C | 50^o^C | 60^o^C | 70^o^C | 50^o^C | 60^o^C | 70^o^C | 50^o^C | 60^o^C | 70^o^C |
| **A** | **Alcohols** |  |  |  |  |  |  |  |  |  |  |  |  |  |
| 1 | 1-Hexanol, 2-ethyl- | 12.7 | - | - | - | 0.0080 | - | - | - | - | - | - | - |  |
| 2 | 2,3-Butanediol | 2.78 | - | - | 0.1413 | - | - | 0.3243 | 0.3243 | 0.5431 | 0.1382 | 0.0009 | - | 0.0023 |
| 3 | 3-Heptanol, 6-methyl- | 12.35 | - | - | - | - | - | - | - | 0.0176 |  |  | - | - |
| 4 | 2,4,7,9-Tetramethyl-5-decyn-4,7-diol | 30.03 | - | - | - | - | - | - | - | 0.0052 | 0.0045 | 0.0007 | - | - |
| **B** | **Acids** |  |  |  |  |  |  |  |  |  |  |  |  |  |
| 5 | Butyric acid, 1-propylpentyl ester | 25.4 | - | - | - | - | - | - | - | - | - | 0.0002 | - | - |
| **C** | **Aldehydes** |  | - | - | - | - | - |  |  |  |  |  | - | - |
| 6 | Benzeneacetaldehyde | 13.08 | - | - | - | 0.0065 | - | 0.0078 | 0.0078 | - | - | - | - | - |
| 7 | 2-Octenal, (E)- | 13.96 | - | - | - | 0.0042 | - | - | - | - | - | - | - | - |
| 8 | Nonanal | 16.31 | - | - | - | 0.0149 | - | 0.0195 | 0.0195 |  | 0.0047 | - | - | - |
| 9 | Decanal | 21.23 | - | - | - | 0.0140 | 0.0176 | 0.0183 | 0.0183 | 0.0109 | 0.0043 | - | - | - |
| 10 | 2-Octenal, 2-butyl- | 28.39 | - | -- | - | 0.0097 | 0.0187 | 0.0190 | 0.0190 | 0.0119 | 0.0060 | 0.0004 | 0.0021 | 0.0025 |
| 11 | Tetradecanal | 37.59 | - | - | - | - | - | 0.0056 | 0.0056 | - | - | - | - | - |
| **D** | **Alkane** |  |  |  |  |  |  |  |  |  |  |  |  |  |
| 12 | Propane, 1-methoxy-2-methyl- | 1.78 | 0.0112 |  | 0.2001 | - | - | - | - | - | 0.1274 | - | - | - |
| 13 | Dodecane | 21.06 | 0.0031 | 0.0064 | 0.0895 | - | 0.1867 | 0.2210 | 0.2210 | 0.2006 | 0.0620 | 0.0019 | 0.0151 | 0.0175 |
| 14 | Tetradecane | 29.38 | 0.0004 | - | 0.0105 | - | 0.0345 | 0.0303 | 0.0303 | 0.0211 | 0.0093 | 0.0005 | 0.0030 | 0.0034 |
| 15 | Cyclopentane, nonyl- | 30.74 | - | - | - | - | 0.0100 | 0.0067 | 0.0067 | 0.0051 | - | - | - | - |
| 16 | Hexadecane | 34.95 | - | - | - | - | 0.0123 | - | - | 0.0066 | - | 0.0002 | - | - |
| 17 | Cyclopentasiloxane, decamethyl- | 19.45 | - | - | - | - | - | 0.0088 | 0.0088 | - | - | 0.0002 | - | - |
| 18 | Pentadecane | 32.36 | - | - | - | - | - | 0.0038 | 0.0038 | - | - | - | - | - |
| 19 | Octacosane | 34.2 | - | - | - | - | - | 0.0048 | 0.0048 | - |  | - | - | - |
| 20 | Cyclotetradecane | 30.73 | - | - | - | - | - | - | - | - | - | - | - | - |
| 21 | Hexadecane | 34.94 | - | - | - | - | - | 0.0089 | 0.0089 | - | - | - | 0.0014 | - |
| 22 | Hexane, 1-ethoxy- | 14.53 | - | - | - | - | - | - | - | - | 0.0108 | - | - | - |
| 23 | Butane, 1,1'-sulfonylbis- | 30.92 | - | - | - | - | - | - | - | - | - | 0.0005 | - | - |
| **E** | **Alkenes** |  |  |  |  |  |  |  |  |  |  |  |  |  |
| 24 | 3-Tetradecene, (Z)- | 29.1 | - | - | - | 0.0048 | - | - | - | - | - | - | - | - |
| 25 | 1-Tetradecene | 29.09 | - | - | - | - | - | 0.0072 | 0.0072 | - | - | - | - | - |
| 26 | 3-Ethyl-3-hexene | 14.65 | - | - | - | - | - | - | - | - | - | - | 0.0203 | - |
| 27 | 13-Methyl-17-norabieta-7,15-diene | 41.88 | - | - | - | - | - | - | - | - | - | - | - | 0.0010165 |
| 28 | Phenanthrene, 7-ethenyl-1,2,3,4,4a | 41.87 | - | - | - | - | 0.0089 | 0.0038 | 0.0038 | - | - | 0.0002 | - | - |
| **F** | **Alkyloids** |  |  | - | - | - | - | - | - | - | - | - | - | - |
| 29 | Dodecane | 20.87 | - | - | - | 0.1743 | - | - | - | - | - | - | - | - |
| 30 | Tridecane | 25.55 | - | - | - | 0.0054 | - | - | - | - | - | - | - | - |
| 31 | Cyclohexasiloxane, dodecamethyl- | 27.2 | - | - | - | 0.0085 | - | - | - | - | - | - | - | - |
| 32 | Tetradecane | 29.38 | - | - | - | 0.0174 | - | - | - | - | - | - | - | - |
| 33 | Cyclotetradecane | 30.73 | - | - | - | 0.0037 | - | - | - | - | - | - | - | - |
| 34 | Hexadecane | 34.95 | - | - | - | 0.0047 | - | - | - | - | -- | - | - | - |
| **G** | **Ester** |  | - | - | - | - | - | - | - | - | - | - | - | - |
| 35 | Hexanedioic acid, bis(2-ethylhexyl) ester | 50.29 | 0.0021 | - | - | - | - | 0.0086 | - | - | - | - | - | - |
| 36 | Hexanoic acid, 4-octyl ester | 31.9 | - | - | - | - | - | - | - | - | - | - | 0.0038 | - |
| 37 | Hexadecanoic acid, ethyl ester | 43.33 | - | - | - | - | - | - | 0.0086 | - | 0.0029 | 0.0002 | 0.0012 | - |
| 38 | Butyric acid, 1-propylpentyl ester | 25.4 | - | - | - | - | - | - | - | - | - | - | 0.0035 | 0.0055 |
| **H** | **ketone** |  |  |  |  |  |  |  |  |  |  |  |  |  |
| 39 | 3-Octanone | 9.64 | 0.0256 | 0.1471 | 0.2499 | 0.2897 | 0.0653 | 0.3059 | 0.3059 | - | 0.1449 | 0.0231 | 0.2192 | 0.2287 |
| 40 | Acetophenone | 14.13 | - | - | - | 0.0081 | - | - | - | - | - | - | - | - |
| 41 | 2-Nonanone | 15.75 | - | - | - | 0.0116 | - | 0.0213 | 0.0213 | 0.0211 | - | 0.0004 | - | 0.0030 |
| 42 | 2-Undecanone | 25.29 | - | - | - | 0.0042 | - | 0.0082 | 0.0082 | 0.0053 | - | - | - | - |
| 43 | 5,9-Undecadien-2-one, 6,10-dimethyl-, (Z)- | 30.99 | - | - | - | 0.0052 | - | - | - | - | - | - | - | - |
| 44 | 2-Tridecanone | 32.26 | - | - | - | 0.0107 | 0.0214 | 0.0190 | 0.0190 | 0.0117 | 0.0066 | 0.0005 | - | - |
| 45 | Ethanone, 1-(2-hydroxy-5-methylphenyl)- | 25.94 | - | - | - | - | - | 0.0144 | 0.0144 | - | - | - | - | - |
| 46 | 5,9-Undecadien-2-one, 6,10-dimethyl-, (E)- | 30.98 | - | - | - | - | - | 0.0078 | 0.0078 | - | - | - | - | - |
| 47 | 2-Dodecanone | 32.27 | - | - | - | - | - | - | - | - | - | - | - | 0.0033 |
| **I** | **Phenol** |  |  |  |  |  |  |  |  |  |  |  |  |  |
| 48 | 2-Methoxy-4-vinylphenol | 25.99 | 0.0006 | - | 0.0135 | 0.0117 | 0.0190 | - | - | 0.0209 | 0.0051 | 0.0002 | - | 0.0036 |
| 49 | Phenol, 2-methoxy- | 32.75 | - | 0.0196 | 0.0196 | 0.0155 | 0.0259 | - | -- | - | 0.0108 | - | - | - |
| 50 | Phenol, 4-ethyl-2-methoxy- | 24.42 | - | - | - | 0.0107 | - | - | - | - | - | - | - | - |
| 51 | Butylated Hydroxytoluene | 32.63 | - | - | - | 0.0036 | 0.0108 | - | - | - | - | 0.0003 | - | - |
| 52 | Phenol, 2,4-bis(1,1-dimethylethyl)- | 32.75 | - | - | - | 0.0066 | 0.0115 | 0.0104 | 0.0104 | 0.0046 | 0.0029 | 0.0005 | 0.0033 | 0.0033 |
| 53 | Phenol, 2,4,6-tris(1,1-dimethylethyl)- | 35.11 | - | - | - | - | - | - | - | 0.0065 | 0.0087 | 0.0001 | - | - |
| **J** | **Pyrazines** |  |  |  |  |  |  |  |  |  |  |  |  |  |
| 54 | Pyrazine, tetramethyl- | 15.27 | 0.0024 | - | - | 0.0400 | - | 0.0672 | 0.0672 | 0.0784 | 0.0136 | 0.0006 | 0.0036 | 0.0071 |
| 55 | 2,3,5-Trimethyl-6-ethylpyrazine | 18.99 | - | - | - | 0.0092 | - | 0.0177 | 0.0177 | 0.0087 | - | - | - | - |

References

[1] A. El-Beltagy, G.R. Gamea, A.H.A. Essa, Solar drying characteristics of strawberry, J. Food Eng. 78 (2007) 456–464. https://doi.org/10.1016/j.jfoodeng.2005.10.015.

[2] E.O.M. Akoy, Experimental characterization and modeling of thin-layer drying of mango slices, Int. Food Res. J. 21 (2014) 1911–1917. https://pdfs.semanticscholar.org/eee4/569452a709c33a45b20e4a48a06d6f8803cf.pdf (accessed December 29, 2018).

[3] A.S. Olawale, S.O. Omole, Thin layer drying models for sweet potato in tray dryer, Agric. Eng. Int. CIGR J. 14 (2012) 131–137. http://cigrjournal.org/index.php/Ejounral/article/view/2060 (accessed July 22, 2018).

[4] N. Hashim, O. Daniel, E. Rahaman, A Preliminary Study: Kinetic Model of Drying Process of Pumpkins (Cucurbita Moschata) in a Convective Hot Air Dryer, Agric. Agric. Sci. Procedia. 2 (2014) 345–352. https://doi.org/10.1016/j.aaspro.2014.11.048.

[5] M.S. Zenoozian, H. Feng, S.M.A. Razavi, F. Shahidi, H.R. Pourreza, Image analysis and dynamic modeling of thin-layer drying of osmotically dehydrated pumpkin, J. Food Process. Preserv. 32 (2008) 88–102. https://doi.org/10.1111/j.1745-4549.2007.00167.x.

[6] K. Kulwinder, S. A.K., Drying kinetics and quality characteristics of beetroot slices under hot air followed by microwave finish drying, African J. Agric. Res. 9 (2014) 1036–1044. https://doi.org/10.5897/AJAR2013.

[7] M. Campus, A.- Bellevue, Mathematical modeling of thin layer drying kinetics of apples slices, Int. Food Res. J. 19 (2006) 1949–1958. https://doi.org/10.1051/IUFoST.

[8] K. Sacilik, Effect of drying methods on thin-layer drying characteristics of hull-less seed pumpkin (Cucurbita pepo L.), J. Food Eng. 79 (2007) 23–30. https://doi.org/10.1016/j.jfoodeng.2006.01.023.

[9] H.M. Thao, A. Noomhorm, Modeling and Effects of Various Drying Methods on Sweet Potato Starch Properties, Walailak J. 8 (2011) 139–158.

[10] C.L. Hii, C.L. Law, M. Cloke, Modeling using a new thin layer drying model and product quality of cocoa, J. Food Eng. 90 (2009) 191–198. https://doi.org/10.1016/j.jfoodeng.2008.06.022.

[11] K. An, D. Zhao, Z. Wang, J. Wu, Y. Xu, G. Xiao,. Comparison of different drying methods on Chinese ginger (Zingiber officinale Roscoe): Changes in volatiles, chemical profile, antioxidant properties, and microstructure, Food Chem. 77 (2016) 4716–4724. https://doi.org/10.1016/j.phytochem.2015.07.012.

[12] P.L. Gan, P.E. Poh, Investigation on the Effect of Shapes on the Drying Kinetics and Sensory Evaluation Study of Dried Jackfruit, Int. J. Sci. Eng. 7 (2014) 193–198. https://doi.org/10.12777/ijse.7.2.193-198.

[13] İ. Doymaz, S. Karasu, M. Baslar, Effects of infrared heating on drying kinetics, antioxidant activity, phenolic content, and color of jujube fruit, J. Food Meas. Charact. 10 (2016) 283–291. https://doi.org/10.1007/s11694-016-9305-4.

[14] A.O. Omolola, A.I.O. Jideani, P.F. Kapila, Modeling microwave drying kinetics and moisture diffusivity of mabonde banana variety, Int. J. Agric. Biol. Eng. 7 (2014) 107–113. https://doi.org/10.3965/j.ijabe.20140706.013.

[15] W.P. da Silva, C.M.D.P.S. e Silva, F.J.A. Gama, J.P. Gomes, Mathematical models to describe thin-layer drying and to determine drying rate of whole bananas, J. Saudi Soc. Agric. Sci. 13 (2014) 67–74. https://doi.org/10.1016/j.jssas.2013.01.003.
